# Deletion of CTCF sites in the *SHH* locus alters enhancer–promoter interactions and leads to acheiropodia

**DOI:** 10.1038/s41467-021-22470-z

**Published:** 2021-04-16

**Authors:** Aki Ushiki, Yichi Zhang, Chenling Xiong, Jingjing Zhao, Ilias Georgakopoulos-Soares, Lauren Kane, Kirsty Jamieson, Michael J. Bamshad, Deborah A. Nickerson, University of Washington Center for Mendelian Genomics, Yin Shen, Laura A. Lettice, Elizabeth Lemos Silveira-Lucas, Florence Petit, Nadav Ahituv

**Affiliations:** 1 Department of Bioengineering and Therapeutic Sciences, University of California San Francisco, San Francisco, CA USA; 2 Institute for Human Genetics, University of California San Francisco, San Francisco, CA USA; 3 School of Pharmaceutical Sciences, Tsinghua University, Beijing, China; 4 MRC Human Genetics Unit, Institute of Genetics and Molecular Medicine, University of Edinburgh, Edinburgh, UK; 5 Department of Pediatrics, University of Washington, Seattle, WA USA; 6 Department of Genome Sciences, University of Washington, Seattle, WA USA; 7 Brotman-Baty Institute, Seattle, WA USA; 8 Department of Neurology, University of California San Francisco, San Francisco, CA USA; 9 Consultorio Genetica Clinica, Porto Alegre, Brazil; 10 CHU Lille, University of Lille, EA7364 RADEME, Lille, France

**Keywords:** Animal disease models, Disease genetics, Development, Chromatin, Transcriptional regulatory elements

## Abstract

Acheiropodia, congenital limb truncation, is associated with homozygous deletions in the *LMBR1* gene around ZRS, an enhancer regulating *SHH* during limb development. How these deletions lead to this phenotype is unknown. Using whole-genome sequencing, we fine-mapped the acheiropodia-associated region to 12 kb and show that it does not function as an enhancer. CTCF and RAD21 ChIP-seq together with 4C-seq and DNA FISH identify three CTCF sites within the acheiropodia-deleted region that mediate the interaction between the ZRS and the *SHH* promoter. This interaction is substituted with other CTCF sites centromeric to the ZRS in the disease state. Mouse knockouts of the orthologous 12 kb sequence have no apparent abnormalities, showcasing the challenges in modelling CTCF alterations in animal models due to inherent motif differences between species. Our results show that alterations in CTCF motifs can lead to a Mendelian condition due to altered enhancer–promoter interactions.

## Abstract (web summary)

Acheiropodia is associated with homozygous deletions in the *LMBR1* gene around ZRS, an enhancer regulating *SHH* during limb development, but how these deletions lead to this phenotype is unknown. Here the authors use whole-genome sequencing, ChIP-seq, 4C-seq and DNA FISH to show that alterations in CTCF motifs are responsible via altered enhancer–promoter interactions.

## Introduction

Acheiropodia (OMIM 200500) is a rare autosomal recessive disorder associated with bilateral congenital transverse defects of the upper and lower limbs including aplasia of the hands and feet^1^. Genetic analysis of five Brazilian families with acheiropodia, three of which were consanguineous, identified a homozygous deletion encompassing exon 4 of the limb development membrane protein 1 (*LMBR1*) gene to be associated with this phenotype^2^. The deletion was estimated to cover 4–6 kilo base (kb) on either side of this exon. However, no assays were done to fine map the deletion or functionally characterize how it could be causing acheiropodia.

While exon 4 of *LMBR1* was deleted in the individuals with acheiropodia, it is likely not the cause of this phenotype. *LMBR1* is a membrane protein that is ubiquitously expressed^3^ and a 35 kb deletion in mice that encompasses exons 1–3 of this gene did not lead to a limb phenotype^4^. *LMBR1* contains an enhancer within intron 5, named the zone of polarizing activity regulatory sequence (ZRS), that regulates the Sonic Hedgehog (*SHH*) gene during limb development. *SHH* encodes a ligand that plays a major role in the development of several tissues, including the limb^5^. In mice, *Shh* is expressed at the posterior part of the limb buds around embryonic day (E) 10–12^6–8^ and plays a central role in digit patterning and limb outgrowth^9,10^. *Shh* homozygous knockout mice display early lethality with defective axial patterning and limb truncation reminiscent of acheiropodia^10^. In humans, heterozygous pathogenic variants in *SHH* are responsible for a large spectrum of central nervous system malformations without any limb malformation, of which the most severe is holoprosencephaly (OMIM 142945)^11^. Bi-allelic*SHH* disruption has not been described in humans. Mutations in the ZRS, located ~1 Mb away of *SHH*, cause non-syndromic limb malformations in humans, mice and many other species, consisting primarily of preaxial polydactyly due to ectopic *SHH* expression in the limb bud^12–14^. In addition, homozygous deletions encompassing the ZRS lead to acheiropodia in humans and mice^15,16^. Collectively, these results indicate that acheiropodia is likely caused by reduced *SHH* expression during limb development. However, the ZRS is completely intact in the Brazilian individuals with acheiropodia who are homozygous for the *LMBR1* exon 4 deletion, suggesting that other functional units associated with *SHH* limb expression may be disrupted by this deletion.

The architectural protein CCCTC-binding factor (CTCF) is known to play a central role in chromatin conformation^17^. It is involved in forming topologically associating domain (TAD), regions in the genome that are on average ~880 kb in length and are defined as having more frequent interactions within this domain than outside it^18,19^. In addition, CTCF is known to mediate long range enhancer–promoter interactions^17^. CTCF-bound sites in a convergent orientation are thought to halt chromatin loops that are progressively being extruded by the cohesin complex^20^, facilitating specific chromatin interaction. Previous studies in mice deleted individual and combinations of CTCF sites in the *Shh* locus, some of them affecting interactions between the ZRS and *Shh* promoter and leading to a reduction of up to ~52% of *Shh* expression in the limb, but none of which led to an observable limb phenotype^4,21^. Interestingly, ectopic CTCF sites appeared in these CTCF motif knockout mice likely supporting compensatory interactions^21^.

We used whole-genome sequencing (WGS) to fine map the homozygous acheiropodia-associated deletion in one of the probands from the Brazilian families, identifying a 12 kb deletion surrounding *LMBR1* exon 4. Using a mouse transgenic enhancer assay, we show that this 12 kb sequence does not have enhancer activity in the developing limb. Further analyses of this sequence using CTCF and RAD21 ChIP-seq identified three CTCF sites in convergent orientation to *SHH* along with RAD21 binding in this region. ChIP-seq analyses in the homozygous proband found an ectopic CTCF site 27 kb centromeric to the ZRS. Consistent with these alterations of CTCF and RAD21 binding, interactions between the *SHH* promoter and the ZRS were found to be impaired in the proband using 4C-seq and DNA fluorescence in situ hybridization (FISH). Finally, we generated a mouse knockout of the orthologous 12 kb acheiropodia-associated region and did not find any limb malformations, highlighting the differential chromatin interactions in this locus in mice compared to humans. Combined, our results suggest that, in humans, CTCF sites adjacent to the ZRS are likely needed as a scaffold to associate the *SHH* promoter to the ZRS and that this mechanism is different in mice.

## Results

### Whole-genome sequencing identifies a 12 kb acheiropodia-associated deletion

**Fig. 1:**
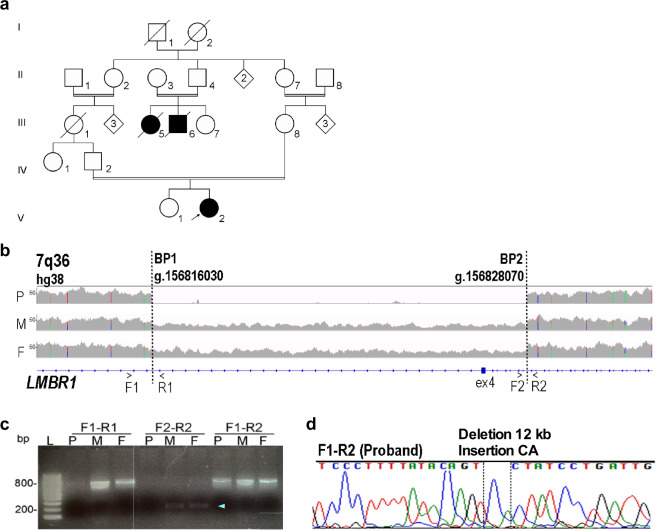
Fine-mapping of the acheiropodia-associated deletion. **a** Pedigree of acheiropodia family with proband indicated via the arrow. Squares and circles represent males and females, respectively. **b** WGS alignments showing a homozygous 12 kb deletion in the acheiropodia proband. The Y-axis is the read depth (number of reads for each nucleotide). The deletion appears in a heterozygous manner in both parents. BP: breakpoint; P: proband; M: mother; F: father. **c** PCR amplification using three different primers pairs, whose location is indicated in **b**, further confirming the breakpoint in the proband (P) and mother (M) and father (F). PCR was performed several times using different primer sets to validate the deletion. **d** Sanger sequencing of the acheiropodia patient showing the breakpoint sequence which also has a CA insertion.

We obtained genomic DNA and lymphoblastoid cell lines from a female proband with acheiropodia and her parents. The proband has terminal transverse hemimelia of the four limbs with truncation of both hands and feet^22^. Prior genetic testing identified a deletion overlapping exon 4 of the *LMBR1* gene with an estimate for the deletion’s boundaries to be around 1.2–2.5 kb and 2.7–3.5 kb 5′ and 3′ of exon 4, respectively^2^. To identify the exact deletion coordinates and assess whether other pathogenic variants might explain the phenotype, we carried out WGS on the proband and her parents. Because of known consanguinity (Fig. 1a), we searched for regions of homozygosity in the proband, finding runs spanning a total of 302 mega base (Mb) within the genome (Supplementary Table 1). Previous genomic analyses of five consanguineous families with acheiropodia, including this family (Family 2 in ref. ^2^), found that all of them share a ~0.5 Mb region of homozygosity in the *LMBR1* gene locus. Based on these results and the known deletion of exon 4, we focused our analyses on this region, identifying a 4 Mb region of homozygosity from rs12719966 to rs1985369 (chr7:155,356,342- 159,326,530; hg38). No pathogenic or likely pathogenic variants were found in *SHH*. In the proband, we identified a 12,041 base pair (bp) homozygous deletion (chr7:156,816,030-156,828,070; hg38) that overlaps *LMBR1* exon 4 along with two base pairs (CA) that were inserted at the breakpoint (Fig. 1b–d, Supplementary Fig. 1). Both unaffected parents are heterozygous for this deletion (Fig. 1b, c, Supplementary Fig. 1). We reported this deletion in the Decipher database^23^ (#411659) and did not identify any overlapping homozygous deletions in control databases^24^. To further validate our WGS results, we carried out both PCR analyses around the breakpoint (Fig. 1c) and Sanger sequencing of the breakpoint (Fig. 1d, Supplementary Fig. 1), the results of which corroborated our findings.

As homozygous deletions of the ZRS, which regulates *SHH* expression in the developing limb, were shown to lead to truncated limbs in mice and humans^15,16^, we carried out detailed sequence analysis of this enhancer. WGS and Sanger sequencing analyses of the ZRS (chr7:156,790,916-156,792,095; hg38) in the proband affected with acheiropodia did not reveal any rare variants in this enhancer. We did observe a homozygous single nucleotide polymorphism (SNP) rs10254391 (chr7:156,791,873; hg38) in the proband and that was heterozygous in both parents. As this SNP has a minor allele frequency of 0.26 in the global population, has been reported to be homozygous in around 1702 cases in GnomAD^25^ and is thought to be benign based on the ClinVar database^26^, we concluded that it is not likely to be causative of this phenotype. Our results strongly suggest that the acheiropodia in the proband is likely caused by the 12 kb homozygous deletion.

### The 12 kb deleted region does not function as a limb developmental enhancer

**Fig. 2:**
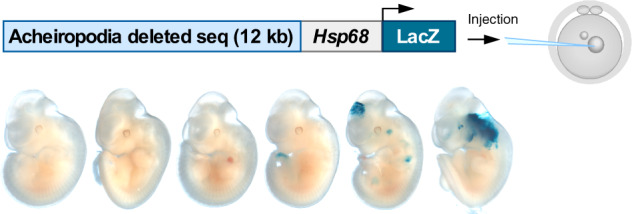
Mouse transgenic enhancer assay for the 12 kb acheiropodia-associated sequence. Schematic representation of the mouse transgenic enhancer assay (upper panel) showing the 12 kb acheiropodia-associated sequence cloned upstream of the *Hsp68* promoter-LacZ gene. Enhancer activity, as visualized by LacZ staining, was not observed in the ZPA for the six PCR positive E11.5 mouse embryos (lower panel).

To test whether this region functions as a developmental limb enhancer, we tested its ability to drive limb expression in mouse embryos. We amplified this 12 kb sequence from a human BAC (RP11-155D20), cloned it into the *Hsp68*-LacZ vector, that contains an *Hsp68* minimal promoter followed by the LacZ reporter gene^27^, and injected it into one-cell mouse embryos (Fig. 2). Transgenic embryos were harvested at E11.5, a time point that is critical for *Shh* limb expression in the zone of polarizing activity (ZPA^6,16^). We obtained six LacZ PCR positive embryos, three not showing any LacZ expression whatsoever and three having inconsistent LacZ expression, none of which have expression in the ZPA (Fig. 2). Previous studies have tested ZRS human sequences/mutations in mice using this assay, finding LacZ expression in the ZPA^28,29^. We also checked this 12 kb region for the presence of various histone modifications indicative of enhancer activity from ENCODE^30^ genomic data. Analysis of 18 different cell types found only a poised enhancer mark, H3K4me1, in two of the cell lines, K562 and A549 (Supplementary Fig. 2). Combined, these results suggest that this 12 kb region does not function as an enhancer in general and more specifically in the ZPA at E11.5.

### The 12 kb deletion leads to altered CTCF/RAD21 distribution

**Fig. 3:**
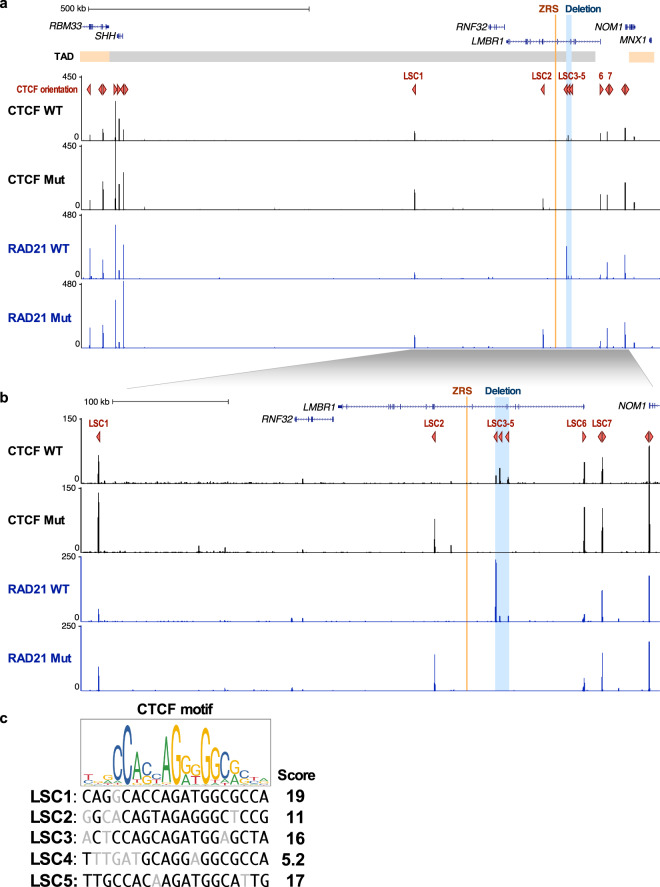
CTCF and RAD21 distribution in the *LMBR1-SHH* locus. **a** CTCF and RAD21 ChIP-seq enrichment in lymphoblastoid cells from wildtype (WT) and proband (Mut) at the *LMBR1-SHH* locus. GM12878 (lymphoblastoid cell line) TAD boundaries are shown in orange and gray horizontal bar. ZRS and the acheiropodia-associated deleted region are shown in orange and blue vertical lines respectively. CTCF orientations are shown as red triangles. The *Y*-axis is the signal *P*-value to reject the null hypothesis that the signal at that location is present in the control. **b** Zoom in of the region around the *LMBR1* gene. **c** CTCF motif from JASPAR^63^ [http://jaspar.genereg.net/] and CTCF motif scores, as assigned by FIMO^33^, overlapping CTCF peaks in the *LMBR1* locus.

We next analyzed the 12 kb deleted region for potential functional entities that could lead to the acheiropodia phenotype. While it overlaps exon 4 of the *LMBR1* gene, mouse knockouts of this gene do not have any apparent limb phenotype^4^, the numerous mutations that were identified in it in humans and mice are thought to lead to limb malformations due to altering ZRS copy number or sequence^12^ and an acheiropodia phenotype was observed in both *Shh* and ZRS homozygous mouse knockouts^10,16^ and homozygous ZRS deletion in humans^15^. We reasoned that the likely cause of the acheiropodia in this proband is altered *SHH* expression during limb development. Analysis of ENCODE^30^ChIP-seq datasets identified three CTCF-bound sites in this region, named here as *LMBR1*-*SHH* CTCF (LSC) sites 3-5. These three CTCF-bound sites appear in numerous ChIP-seq assays (LSC3: 118/191, LSC4: 97/191, LSC5: 139/191) from various human cell lines, strongly suggesting that they are functional (Supplementary Fig. 3, Supplementary Table 2). As CTCF motif orientation was shown to be important in determining the positioning of chromatin looping^31^, we next analyzed the orientation of these sites. We found that all three sites are in convergent orientation to the *SHH* gene (Fig. 3a). We thus speculated that this 12 kb region may function as a scaffolding region, enabling ZRS to interact with the *SHH* promoter.

To test whether this sequence functions as a scaffolding region, we carried out ChIP-seq for both CTCF and RAD21, a member of the cohesin complex that along with CTCF is known to determine chromatin looping^32^. ChIP-seq was done for both proteins using proband and wild-type lymphoblastoid cells. It is important to note that these cells were established using an Epstein Barr virus which could affect our subsequent genomic studies. As a previous study^21^ indicated that the interaction between *Shh* and ZRS is “tissue-invariant”, we reasoned that these cells could be used for these analyses. We also checked the mRNA expression of *SHH* in the wild-type and mutant cells, observing overall low expression levels that were significantly higher in wild-type versus proband cells (Supplementary Fig. 4). In the wild-type cells, we observed three CTCF ChIP-seq peaks (LSC3-5) that have sites in convergent orientation to *SHH* and correspond to those found in the ENCODE datasets (Fig. 3a, b, Supplementary Figs. 5, 6). For RAD21, we also observed binding in the 12 kb region, in particular at the LSC3 site (Fig. 3a, b, Supplementary Figs. 5, 6). In the proband’s cells, we did not observe the CTCF and RAD21 peaks due to the 12 kb deletion. Instead, we observed a novel RAD21 and CTCF peak in convergent orientation to *SHH* (LSC2) near exon 6 of *LMBR1* that does not appear in wild-type cells (Fig. 3a, b, Supplementary Figs. 5, 6).

We next analyzed the CTCF motif scores of LSC1-5 to assess whether they could be associated with the appearance of the novel CTCF binding (LSC2) observed in the proband’s cell line. We used the Find Individual Motif Occurrences (FIMO^33^) tool to assign motif scores for all five sites. We extracted the CTCF motifs with a *P*-value threshold of 0.001 genome-wide and only picked motifs in the *SHH*-*LMBR1* locus that overlapped CTCF peaks in our ChIP-seq. For LSC3-5, we observed motif scores, determined by the weights at the corresponding position-weight matrix summing up to 16, 5.2, and 17 respectively, while for LSC2 we obtained a score of 11 (Fig. 3c). This suggests that with the loss of LSC3-5 due to the deletion, CTCF might bind to the LSC2 weaker binding affinity motif instead of LSC3-5.

### The 12 kb deletion impairs the interaction between ZRS and the *SHH* promoter

**Fig. 4:**
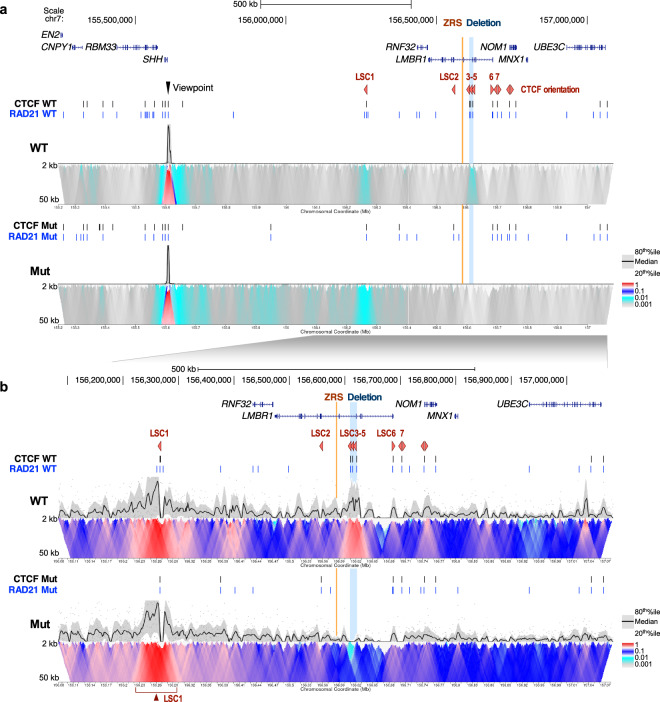
Chromatin interactions with the *SHH* promoter. **a** 4C contact profiles in lymphoblastoid cells from wildtype (WT) and proband (Mut) at the *LMBR1*-*SHH* locus. The viewpoint is depicted by a black arrowhead. The median and 20th and 80th percentiles of sliding 2–50 kb windows determine the main trend line. The color scale represents enrichment relative to the maximum medium value attainable at 12 kb resolution. CTCF and RAD21 ChIP-seq peaks are shown as black and blue vertical line respectively. The ZRS and the acheiropodia-associated deleted region are shown as orange and blue vertical lines respectively. CTCF orientations are shown as red triangles. **b** Zoom in of the region around the *LMBR1* gene.

To examine whether the chromatin interaction between the ZRS and the *SHH* promoter is altered due to the 12 kb deletion, we performed 4C-seq using the *SHH* promoter as a viewpoint. 4C-seq was performed on both proband and wild-type lymphoblastoid cell lines using standard methods^34^ (see “Methods”). In wild-type cells, we observed that the *SHH* promoter strongly interacts with LSC1 and LSC3-5 (Fig. 4a, Supplementary Figs. 5, 6). For the proband’s cells, we did not observe interactions with the ZRS and instead saw increased interactions between the *SHH* promoter and LSC1 (Fig. 4b, Supplementary Figs. 5, 6). Interestingly, in wild-type cells we observed a weak interaction with the ZRS compared to a much stronger interaction between LSC3-5 and the *SHH* promoter (Fig. 4b, Supplementary Figs. 5, 6). We also analyzed published CTCF Hi-ChIP data from human GM12878 lymphoblastoid cells^35^ and observed a much more robust interaction between the *SHH* promoter and the 12 kb region compared to the ZRS (Supplementary Fig. 7).

**Fig. 5:**
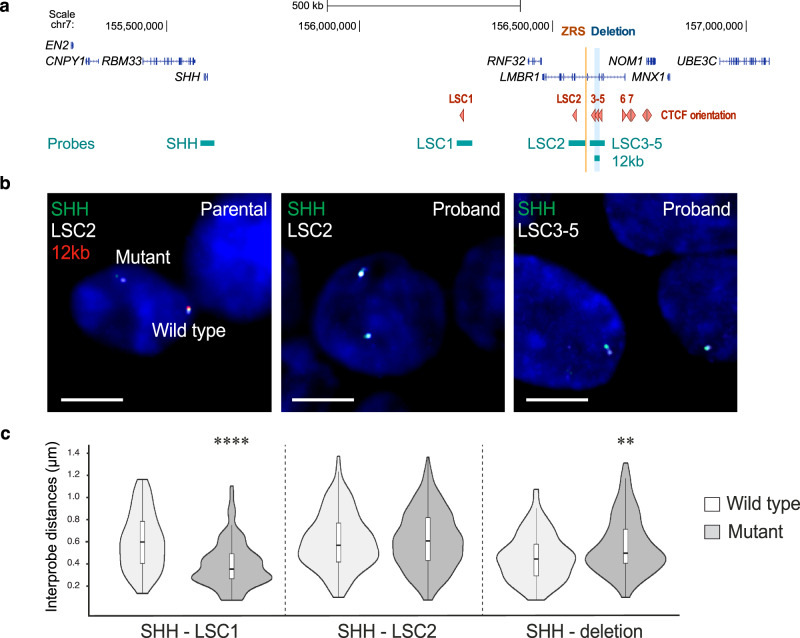
DNA FISH showing the *SHH* promoter interaction with the acheiropodia-associated region. **a** Schematic of the *LMBR1-SHH* locus showing the ZRS and the acheiropodia-associated deleted region via orange and blue vertical lines respectively. CTCF orientations are shown as red triangles and the locations to which the DNA FISH probes hybridize to are depicted by light blue bars. **b** Images of representative nuclei from DNA FISH analysis of parental and proband lymphoblastoid cells showing FISH signals for SHH, LSC2, LSC3-5, and 12 kb probes. Scale bars: 5 µm. **c** Violin plots showing the distribution of interprobe distances (µm) between SHH – LSC1, SHH – LSC2, and SHH – Deletion. The wild-type allele was distinguished from the mutant allele in the parental cell line using the 12 kb probe. SHH – deletion is measured from SHH to 12 kb probe on the wild-type allele and from SHH to LSC3-5 probe on the mutant allele. The statistical significance between datasets was examined by a two-sided Mann–Whitney U-test, ** = 0.004537 and **** = 2.083×10^−11^ (*n* = 75–150 alleles).

DNA FISH was also carried out on both proband and parental lymphoblastoid cell lines to investigate chromosome conformation changes in an allele-specific manner using probes targeting the *SHH* promoter, LSC1, LSC2, and LSC3-5 (Fig. 5a). To distinguish between the wild-type and mutant alleles in the parental cell lines, we used a plasmid containing the 12 kb acheiropodia-associated region (Fig. 5b). LSC1 was found to be significantly closer to the *SHH* promoter on the mutant allele compared to the wild-type chromosome, suggestive of an increased interaction between LSC1 and the *SHH* gene. We also observed that the novel LSC2 peak identified in the proband’s cell line is not found closer to the *SHH* promoter when comparing the wild-type allele to the mutant (Fig. 5c). However, we did observe a significant increase in the distance between the *SHH* promoter and the region containing the 12 kb deletion that was specific to the mutant allele consistent with the loss of interactions observed by 4C-seq (Fig. 4). These results further suggest that the 12 kb acheiropodia-associated region functions as a scaffolding region between the ZRS and the *SHH* promoter and its deletion impairs this interaction.

### Removal of the acheiropodia-associated region in mice does not lead to an observable phenotype

**Fig. 6:**
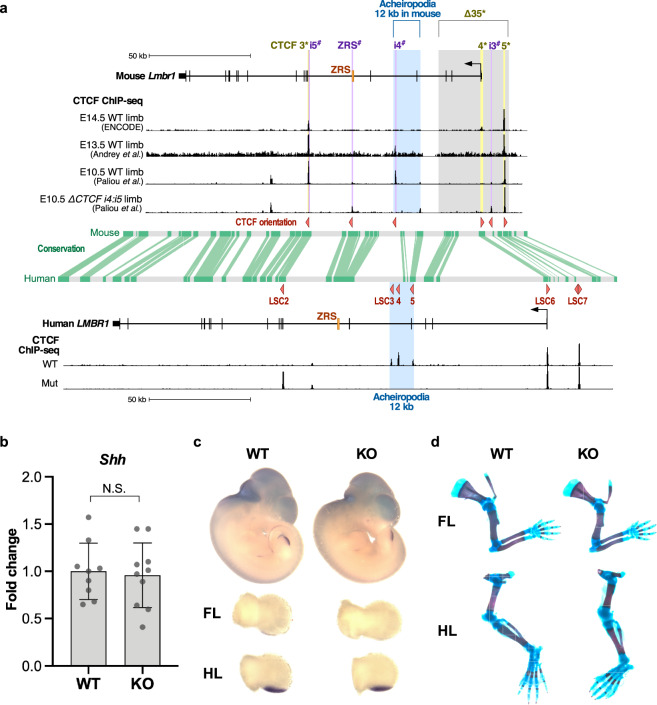
Human and mouse genomic comparisons and phenotype of mice where the orthologous region was deleted. **a** Comparison of the *LMBR1-SHH* locus between human and mice. CTCF site deletions analyzed by Paliou et al.^21^ are marked by purple lines and those generated by ^#^, Williamson et al.^4^ are denoted by yellow lines or gray rectangle, and marked by *. CTCF motif orientation is shown via red triangles. The acheiropodia-associated deletion and its mouse orthologous sequence are depicted by a blue rectangle. Mouse limb CTCF ChIP-seq data from ENCODE^37^, Andrey et al.^38^, Paliou et al.^21^ and human CTCF ChIP-seq data from this study (WT = wild type; Mut=proband) are shown as black genomic tracks below the locus. The conservation track is adopted from the Ensembl Genome Browser^71^ with green lines indicating conserved sequences between humans and mice. **b** *Shh* gene expression levels dissected from E11.5 mouse autopods from wild type (WT) and knockout (KO) mice as determined by qRT-PCR. Each value represents the ratio of *Shh* gene expression to that of *β-Actin*, and values are mean ± standard deviation. The expression value of WT group was arbitrarily set at 1.0. Each dot represents one embryo and statistical differences were determined using a two-sided unpaired *t* test (*P* = 0.7796, N.S., not significant). Source data are provided as a Source Data file. **c** Whole-mount in situ hybridization for *Shh* of wild type (WT) and knockout (KO) E11.5 mouse embryos. Forelimbs (FL) and hindlimbs (HL) were dissected and shown in the lower panel. **d** Wild type (WT) and knockout (KO) E18.5 limb skeletal staining using alizarin red/alcian blue.

To further assess the function of this sequence in mice, we generated a mouse knockout of the orthologous 12 kb acheiropodia-associated region. Using the liftOver tool in the UCSC Genome Browser^36^ (see “Methods”) the human 12 kb acheiropodia-associated sequence was converted to its orthologous mouse sequence (chr5:29,335,354-29,348,393; mm10). Of note, using FIMO^33^ we observed that mice have eight CTCF motifs in this orthologous region while humans have four, and only one of the eight overlapped mouse limb CTCF ChIP-seq data^37,38^ (Fig. 6a). We also analyzed developing mouse embryonic limb (E10.5-E15.5) ChIP-seq datasets for various histone modifications (H3Kme1, H3K4me2, H3K4me3, H3K9ac, H3K9me3, H3K27ac, H3K27me3, H3K36me3) and ATAC-seq from ENCODE^30^ and did not observe any peaks overlapping this region (Supplementary Fig. 8). Previous deletions of various CTCF sites in this region in mice did not show any apparent limb malformations^4,21^. However, these deletions did not cover the acheiropodia-associated region (Fig. 6a). We generated a knockout mouse which harbors the orthologous 12 kb acheiropodia-associated deletion along with additional sequence due to sgRNA selection constraints (chr5:29,334,962-29,348,393; mm10). Mouse knockouts were generated using the improved-Genome editing via Oviductal Nucleic Acids Delivery (*i*-GONAD^39^) technique. Founder mice and germ line transmission in F1 offspring with the desired deletion were validated by PCR, Sanger sequencing and Southern blot (Supplementary Fig. 9). We focused our subsequent phenotypic analyses on mouse line 517 that had a single nucleotide T insertion within the deleted region (Supplementary Fig. 9a).

To determine the functional effect of the deletion, we generated homozygous mice and phenotyped them using qRT-PCR, whole-mount in situ hybridization (WISH) and alizarin red/alcian blue skeletal staining. Homozygous mice did not have any observable phenotype. qRT-PCR on E11.5 autopods from both forelimbs and hindlimb did not identify *Shh* expression changes between homozygous and wild-type mice (Fig. 6b). WISH for *Shh* did not identify any changes in expression between homozygous and wild-type E11.5 embryos (Fig. 6c). Finally, we checked the limb skeletal structure at E18.5 using alizarin red/alcian blue staining finding no apparent abnormalities in the homozygous embryos (Fig. 6d). These results highlight that mice are not an appropriate model to test the chromosomal interactions in humans for this region, likely due to the differences in CTCF site distribution and orientation. In addition, they also suggest that removal of *Lmbr1* exon 4 does not lead to a limb-associated phenotype in mice.

## Discussion

**Fig. 7:**
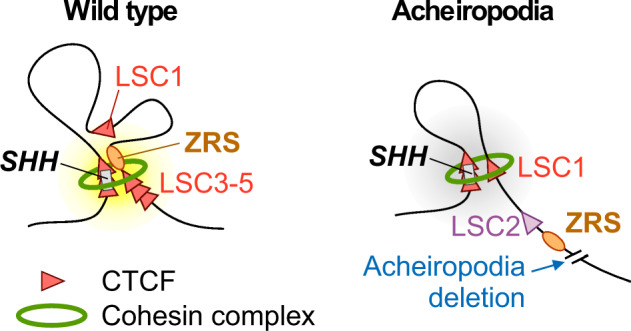
Proposed model for the aberrant chromatin structure of the *LMBR1*-*SHH* locus in the acheiropodia patient. Model of chromatin structure in the *LMBR1*-*SHH* locus based on our ChIP-seq and 4C-seq data. CTCF sites are shown as red triangles and the cohesin complex is shown as a green ring. The ZRS is depicted as an orange oval.

We identified a 12 kb homozygous deletion that is associated with acheiropodia. We show that this 12 kb region does not have enhancer activity at mouse E11.5. Our CTCF and RAD21 ChIP-seq data indicate that this region has three CTCF binding sites along with RAD21 binding (Fig. 3b). Chromatin interaction analyses of this region suggests that it functions as a scaffolding region between the ZRS and the *SHH* promoter via three CTCF sites (LSC3-5). In the cells from the proband with acheiropodia, these sites are deleted and this interaction is substituted with another CTCF site (LSC1) centromeric to the ZRS. Due to this change in interaction, the ZRS does not interact with the *SHH* promoter (Fig. 7). Deletion of the orthologous region in mice did not lead to an observable phenotype, likely due to the inherent chromatin interaction and CTCF distribution differences between humans and mice in this region.

Our work suggests there are substantial differences in the regulation of chromosomal interactions linking ZRS to the *SHH* promoter between humans and mice. There are two previous reports that generated CTCF site-specific deletions around the ZRS in mouse^4,21^. Paliou et al.^21^ deleted the i4^#^ or i5^#^ CTCF sites (Fig. 6a) individually or together and observed no major limb phenotype even though a 51% reduction of *Shh* expression was observed in E10.5 limb buds when both CTCF sites were deleted. Following the deletion of these two CTCF sites (i4^#^ and i5^#^), ectopic CTCF sites also appeared, one within the ZRS (ZRS^#^) and the other near the transcription start site (TSS) of *Lmbr1* (termed here as i3^#^), both of which do not overlap our observed ectopic CTCF site, LSC2 (Fig. 6a). To characterize the function of the ectopic CTCF sites, three sites (i4^#^, i5^#^ and ZRS^#^) were deleted, leading to a depletion of all CTCF (including i3^#^) and RAD21 binding around the ZRS and significantly decreasing the interaction between *Shh* and ZRS. Although these triple deletions led to a 52% reduction of *Shh* expression in E10.5 limb buds, no limb abnormalities were observed. Williamson et al.^4^ deleted three different CTCF sites individually around the *Shh* gene and ZRS (CTCF3*, 4*, and 5*; Fig. 6a). Mice homozygous for each deletion did not show an observable limb phenotype. They also generated a homozygous 35 kb deletion that contains CTCF4*, i3^#^, 5* and the *Lmbr1* TSS and promoter and did not observe *Shh* gene expression changes in E11.5 limb buds measured by qRT-PCR and any apparent limb abnormalities. Of note, as previously mentioned, these results also suggest that *Lmbr1* itself is not necessary for limb development, as also observed in our 12 kb knockout mice. Combined, these CTCF mouse deletion studies, including our study, imply that ZRS-*Shh* interactions are likely to be robust to individual or even triple CTCF perturbations. They also suggest that other CTCF sites, either those that were not tested in these studies or ones that appear ectopically following these manipulations, keep this interaction intact.

The phenotypic differences between human and mice are likely due to several factors including differences in CTCF location, motif score and orientation. In terms of location, humans have three CTCF ChIP-seq peaks (LSC3-5) in the deleted region; however, mouse has one CTCF peak in the orthologous 12 kb acheiropodia deleted sequence (i4^#^) and this region does not show strong evolutionary conservation between humans and mice (Fig. 6a). Our work suggests that these three human CTCF sites (LSC3-5) play a role as an anchor/scaffolding region for the interaction between ZRS and the *SHH* promoter. In mice, i4^#^ and i5^#^ likely play this role and their relative distal position between one another might be important for robustness. Analysis of previously published 4C-seq from E10.5 mouse limbs^21^ showed interactions between the *Shh* promoter and i9^#^, CTCF3*/i5^#^ and ZRS^#^ but not with the 12 kb acheiropodia-associated region (Supplementary Fig. 10). These CTCF sites could be working cooperatively to maintain the interaction between ZRS and the *Shh* promoter. For LSC1, where we observed increased interactions with the *SHH* promoter in the proband, CTCF ChIP-seq from ENCODE^30^ has 161/191 assays showing CTCF-bound sites in this region while in ChIP-seq datasets from various mouse tissues/cells we only observed about half of the assays to have a peak in this region (Supplementary Fig. 11). Interestingly, for LSC2, while we only observed a CTCF ChIP-seq peak in the proband, likely due to compensation for the deletion of LSC3-5, in mice the homologous CTCF site, 3*/i5^#^, shows a strong CTCF ChIP-seq peak in wild-type E10.5, E13.5 and E14.5 limbs (Fig. 6a) and in ENCODE^30^ mouse datasets (Supplementary Fig. 11). Analyses of CTCF motif scores for LSC2 versus 3*/i5^#^ shows a weaker score for LSC2 (Supplementary Fig. 12a), and this CTCF site also has a weak interaction with the *SHH* promoter (Fig. 4). Weaker CTCF sites might serve as a backup for failed enhancer–promoter interactions. Correspondingly, analyses of ZRS^#^ and i3^#^ ectopic CTCF sites which appeared in the i4^#^ and i5^#^ double deletion mice found them to have lower motif scores than i4^#^ and i5^#^ (Supplementary Fig. 12b).

In terms of orientation, all three CTCF sites in the 12 kb acheiropodia-deleted region (LSC3-5) are in convergent orientation to *SHH* along with LSC1 and LSC2, while the sites telomeric to LSC5, LSC6 and LSC7, are in divergent orientation or both (Fig. 6a). In mice, CTCF site 4* that is homologous to LSC6 and 5* which is homologous to LSC7 are all in divergent orientation to *Shh*, but i3^#^ is in convergent orientation (Fig. 6a). Carrying out a more global analysis of human and mouse CTCF ChIP-seq peaks that compared human K562 to mouse CH12 cells, both lymphoblasts, shows that only around 25% of the peaks overlap when converting their coordinates to mouse or vice versa (Supplementary Fig. 13). These results are consistent with a recent report that also analyzed the overlap of CTCF ChIP-seq peaks between these cells (K562 and CH12) plus human GM12878 and mouse MEL cells^40^. This suggests that there are major differences between human and mouse in terms of CTCF location. These differences could be due to various selection pressures, proving more safeguards for enhancer–promoter interactions. It will also be intriguing to test whether these changes in CTCF location and orientation could be involved in phenotypic differences between species. Taken together, our results highlight that mouse is not a useful model to assess the chromatin interactions in humans for this locus and that CTCF location, orientation and number needs to be assessed between human and mice before using mice as an animal model to dissect human nucleotide variation that affects CTCF binding.

The 12 kb acheiropodia-associated deleted region resides close to a topologically associated domain (TAD) boundary that encompasses both *SHH* and *LMBR1*. Previous mouse genetic studies have shown that TAD boundary alterations could alter chromatin interactions and lead to ectopic gene expression^31,41^. While we cannot definitively exclude that this deletion is associated with TAD boundary alterations, using the 3D Genome Browser^42^, we have analyzed this TAD boundary in Hi-C datasets from ten different human cell lines, finding that in all of them the boundary does not overlap this 12 kb deleted region. We observed two different locations for this boundary that differ between cell types. For five of the cell lines (HepG2, GM12878, NHEK, K562, and HMEC), this boundary is thought to be located around the *LMBR1* TSS while for five other cell lines (H1-ESC, G401, A549, epidermal keratinocyte and hippocampus) the boundary is estimated to be around the transcription termination site of the DnaJ heat shock protein family (Hsp40) member B6 (*DNAJB6*) gene (Supplementary Fig. 14). Human and mice TAD boundaries were shown to be relatively conserved^43^. In mice, the *Shh*-*Lmbr1* TAD boundary resides around the *Lmbr1* TSS^44^, similar to what is observed in humans for five out of the ten cell lines. Symmons et al.^44^ inverted a 450 kb region (300 kb downstream and 150 kb upstream of *Lmbr1*) that contains this boundary. This led to a complete loss of *Shh* expression in the ZPA and a limb truncation phenotype, similar to the ZRS homozygous knockout. 4C-seq analysis revealed that the ZRS-*Shh* interaction was disrupted in this inversion, further suggesting that altering this interaction can lead to an acheiropodia like phenotype. In summary, while we cannot conclusively rule out that alteration of the TAD boundary is responsible for this phenotype, our results strongly suggested that removal of these CTCF sites in humans alters the interaction between ZRS and the *SHH* promoter, likely leading to the acheiropodia phenotype.

CTCF plays a major role in enhancer–promoter interactions, facilitating transcriptional activity by establishing chromatin loops between these elements^45,46^. However, only a small number of genetic diseases where CTCF site-specific mutations lead to alterations of these enhancer–promoter interactions have been reported. CTCF site-specific deletions were shown to be associated with imprinting in the *IGF2/H19* locus, causing Beckwith-Wiedemann syndrome (BWS; OMIM 130650). A 1.8 kb deletion that removes two CTCF sites in the normal region imprinted and silenced *IGF2* expression in the maternal allele was shown to lead to hypermethylation and biallelic expression of *IGF2* and is thought to cause BWS^47^. Several reports have associated somatic mutations in CTCF sites with various cancers^48,49^. Interestingly, analysis of somatic mutations from the International Cancer Genome Consortium database^50^ revealed that numerous mutations overlap human stem cell CTCF loop anchors^51^, suggesting that aberrant chromatin interactions could be strongly associated with cancer. To our knowledge, this study is the first to report a CTCF mutation that is associated with a Mendelian condition. With WGS becoming more commonly used in the clinic, it would be interesting to analyze disease-associated variants, in particular short indels, for their overlap with CTCF motifs and chromatin interactions. In addition, our study shows that due to differences in CTCF site location, motif sequence and orientation animal models may not be a good proxy to analyze the effects of CTCF site variation. As more human genomes are sequenced and the genomes of additional species become available, it will be important to consider the phenotypic effects of nucleotide changes in CTCF sequences on disease and evolution.

## Methods

### Patient sample collection

The study was approved by the ethical committee of the University of California San Francisco, protocol number 10-03111, Comitê de Ética em Pesquisa da Prefeitura de Porto Alegre (Plataforma Brasil) protocol number 1.103.654 and the Brazilian Research Ethics Commission (CONEP) protocol number 223.811. Samples were obtained after receipt of informed consent. Genomic DNA was extracted from saliva using standard techniques. Blood samples were collected using standard techniques and used for the generation of lymphoblastoid cell lines. Clinical data were obtained from a physician examination and review of medical records.

### Establishment of lymphoblastoid cell line and culture

Blood samples from the proband and parents were spun over Ficoll-Paque (Amersham Biosciences) gradients to enrich the sample for mononuclear cells. Epstein Bar virus (EBV)-transformed lymphoblastoid lines were generated from isolated peripheral blood lymphocytes. Briefly, cells were washed and resuspended in complete Iscove’s modified Dulbecco’s culture media supplemented with 10% v/v fetal bovine serum, antibiotics, and virus. The B95-8 EBV-infected marmoset cell line (ATCC, catalog no. CRL-1612) was used as the source for viral stocks. High molecular weight DNA was isolated from Ficoll-Paque enriched mononuclear cells using standard desalting procedures. Lymphoblastoid cells were maintained in RPMI1640 medium (Life Technologies, catalog no. 11875093) containing 15% fetal bovine serum (FBS) and penicillin-streptomycin.

### Whole-genome sequencing

Whole-genome sequencing was performed at the University of Washington Center for Mendelian Genomics (University of Washington, Seattle). Initial quality control (QC) entailed DNA quantification, gender validation assay, and molecular fingerprinting with a 63-SNP OpenArray assay derived from a custom exome SNP set. Following successful QC, at least 750 ng of genomic DNA was subjected to a series library construction steps utilizing the KAPA Hyper Prep kit (Roche), automated on the Perkin Elmer Janus platform. Libraries were validated using the Bio-Rad CFX384 Real-Time System and KAPA Library Quantification kit (Roche). Samples were sequenced on a HiSeq X using Illumina’s HiSeq X Ten Reagent Kit (v2.5) to an average depth of 30X. Burrows-Wheeler Aligner^52^, Genome Analysis ToolKit^53^ and SeattleSeq Annotation server build 138 (https://snp.gs.washington.edu/SeattleSeqAnnotation138/) were used to generate BAM, vcf and annotation files, respectively. Homozygosity mapping was performed with PLINK v1.07 software^54^ using the genotypes generated by the 63-SNP OpenArray assay. Structural variants were called using Lumpy^55^. Alignments were visualized using the Integrative Genomics Viewer tool^56^. The *LMBR1* deletion and ZRS variants were validated by PCR-Sanger sequencing (primers provided in Supplementary Table 3).

### Mouse transgenic enhancer assays

Mouse work was approved by the UCSF Institutional Animal Care and Use Committee (IACUC), protocol number AN181381, and was conducted in accordance with AALAC and NIH guidelines. The 12 kb acheiropodia-associated region was amplified from a human BAC (RP11-155D20) by PCR, cloned it into the *Hsp68*-LacZ vector^27^ and sequence verified. All LacZ transgenic mice were generated by Cyagen Biosciences using standard procedures^57^, and harvested and stained for LacZ expression at E11.5 as previously described^58^. Pictures were obtained using an M165FC stereo microscope and a DFC500 12-megapixel camera (Leica).

### ChIP-seq

Lymphoblastoid cells were plated on two different flasks and used for the experiment as independent tubes considered as two technical replicates. Cells (1 × 10^7^ cells) were fixed in phosphate-buffered saline (PBS) with 0.96% formaldehyde for 8 min at room temperature. Crosslinking was quenched with 125 mM Glycine. The cells were washed with PBS and precipitated via centrifugation. The cell pellet was stored in −80 °C until use. The pellet was lysed in 240 µL of Buffer B (LowCell# ChIP kit; Diagenode, catalog no. C01010072) and lysed chromatin was sheared using a Covaris S2 sonicator to obtain on average 250 bp size fragments. ChIP was performed using the LowCell# ChIP kit according to the manufacturer’s protocol with modifications. 120 µL of sheared chromatin was mixed with 880 µL of Buffer A (LowCell# ChIP kit) supplemented with complete protease inhibitor (Sigma-Aldrich, catalog no. 11873580001). 80 µL of the solution was saved as input control. To obtain magnetic bead-antibody complexes, 22 µL of protein A-coated paramagnetic beads (LowCell# ChIP kit) were washed twice with Buffer A (LowCell# ChIP kit) and resuspended in 22 µL of Buffer A. 10 µL of magnetic beads were mixed with 90 µL of Buffer A (LowCell# ChIP kit) and 6 µg antibody (final antibody concentration was 60 ng/µL in the binding reaction). This mixture was gently agitated at 4 °C for 2 h. Antibody against CTCF (Active Motif, catalog no. 61311) or RAD21 (Abcam, catalog no. ab992) was used for immunoprecipitation respectively. The bead-antibody complex was precipitated with a magnet and the supernatant was removed. 800 µL of shared chromatin was added to the bead-antibody complex and rotated at 4 °C overnight. The beads were then washed with Buffer A three times and Buffer C once. DNA was purified using IPure kit v2 (Diagenode, catalog no. C03010015) according to the manufacturer’s protocol. Sequencing libraries were generated using the Accel-NGS 2 S Plus DNA Library Kit (Swift Biosciences, catalog no. 21024) according to the manufacturer’s protocol. Massively parallel sequencing was performed on an Illumina HiSeq4000 with 50 bp single-end read. ChIP-seq was done with two technical replicates. ChIP-seq data were analyzed following the ENCODE transcription factor pipeline^59^. Both RAD21 and CTCF ChIP-seq raw reads were mapped against the human genome (GRCh37; hg19) using bowtie2 (v2.2.6). Duplicate reads were marked using Picard (v1.126) MarkDuplicates and multimapping, low quality, duplicated and non-properly paired reads were removed. Library complexity measures and flagstats were generated for each BAM file.

BAM files were converted to tagAlign format and two subsampled pseudoreplicates were generated for each sample with half the total reads. Reproducible peaks were identified using the MASC2 (v2.1.1)^60^ peak caller and the irreproducibility discovery rate (IDR (v2.0.4)) framework^59^. IDR analysis was performed using self-pseudoreplicates and the main samples to obtain self-consistent sets of peaks. Final peak calls were filtered using the ENCODE blacklist^61^ and an IDR of 2% with a signal value > 30. We combined replicates to obtain only highly reproducible peaks using the IDR^59^ and show them pooled in main figure and individual replicates in supplementary figures. Differential enrichment analysis between the proband and wild-type cells was performed by DiffBind^62^.

### CTCF motif analysis

We used the position-weight matrix MA0139.1 from JASPAR^63^ to scan for CTCF motifs. Genome-wide CTCF motif identification was performed on the human (hg19) and mouse (mm9) genomes, with FIMO^33^, with a *P*-value threshold of 0.0001. For some CTCF peaks in the *SHH*-*LMBR1* locus (LSC4, 6, and 7), when there was no CTCF motif that overlapped ChIP-seq peaks, we reduced the *P*-value threshold to 0.001. CTCF orientation was determined by the strand in which the motif was identified. For the human-mouse CTCF ChIP-seq comparisons, experiments from the cell lines K562^30^ and CH12^37^ were analyzed. The UCSC Genome Broswer^36^ liftOver tool was used with –minMatch=0.01 to transfer the peak coordinates between the two species followed by BEDtools^64^ to intersect them and calculate the proportion of overlapping peaks.

### 4C-seq

Lymphoblastoid cells were plated on two different flasks and used for the experiment as independent tubes deemed as two technical replicates. 4C-seq was performed using standard procedures^34^. Briefly, 1 × 10^7^ cells were fixed in PBS with 2% formaldehyde for 10 min at room temperature. Crosslinking was quenched with 125 mM Glycine. The cells were precipitated via centrifugation and resuspended in lysis buffer (50 mM Tris-HCl pH7.5, 150 mM NaCl, 5 mM EDTA, 0.5% NP-40, 1.15% Triton X-100, 1x complete proteinase inhibitors (Roche, catalog no. 11697498001)) and incubated for 10 min on ice. They were then precipitated via centrifugation and washed with PBS. The cell pellet was stored in −80 °C until use. The cell pellet was suspended in *Dpn*II restriction enzyme buffer and treated with 0.3% SDS and 2.5% Triton X100 at 37 °C for 1 h, respectively. Chromatin was digested with 100 units of *Dpn*II (New England Biolabs, catalog no. R0543) at 37 °C for 3 h. An additional 100 unit of *Dpn*II was added and the reaction was incubated at 37 °C overnight. After heat inactivation of the enzyme, 50 units of T4 DNA ligase (Roche, catalog no. 10799009001) were applied for self-ligation of the digested chromatin and placed for incubation at 16 °C overnight. After purification of DNA using phenol-chloroform and ethanol precipitation, DNA was digested with 50 units of *Nla*III (New England Biolabs, catalog no. R0125) at 37 °C overnight. Following heat inactivation of the enzyme at 65 °C, 25 µg of DNA was used for the second ligation reaction with 50 units of T4 DNA ligase at 16 °C overnight. After purification of DNA using phenol-chloroform and ethanol precipitation, the inverse PCR was performed using NEBNext high-fidelity 2X PCR master mix (New England Biolabs, catalog no. M0541). DNA was purified with AMPure XP beads (Beckman Coulter, catalog no. A63881). The second round of PCR was performed using NEBNext high-fidelity 2X PCR master mix to attach library adapters and index sequences. All PCR primer sequences are listed in Supplementary Table 3. DNA was purified with the QIAquick PCR purification kit (Qiagen, catalog no. 28104). Massively parallel sequencing was performed on an Illumina HiSeq4000 with 50 bp single-end reads using a custom primer (Supplementary Table 3). 4C-seq was carried out using two technical replicates. 4C-seq data were analyzed using the 4C-seq pipeline^65^. Briefly, 4C-seq raw reads were trimmed to 50 bp with cutadapt 2.4. Valid 4C-seq reads containing 4 C reading primer were extracted from fastq file and parsed into raw.txt file aligned against the restriction-enzyme digested genome GRCh37(hg19) using 4Cseqpipe version 0.7^65^. Raw files were translated into final graphical depictions of contact profiles around viewpoints using 4Cseqpipe version 0.7^65^.

### DNA FISH

For DNA FISH, 0.5–1 × 10^6^ lymphoblastoid cells were seeded on Poly-prep slides (Sigma) overnight. They were then fixed in 4% paraformaldehyde for 10 min at room temperature and permeabilized using 0.5% Triton X for 10 min^66^. Fosmid clones and plasmid were prepared and labeled as previously described^67^. Cells were denatured for 30 min. For four-color FISH, each slide was hybridized with between 80 and 100 ng of biotin-, digoxigenin- and directly labeled probes, 18 µg of human Cot1 DNA (Invitrogen) and 5 µg salmon sperm DNA. Green496-dUTP (Enzo Life Sciences) was used for direct labeling of fosmid probes. Washes and detection were as previously described^67^. See Supplementary Table 3 for Fosmid probe details.

Slides were imaged using a Photometrics Coolsnap HQ2CCD camera and a Zeiss AxioImager A1 fluorescence microscope with a Plan Apochromat 100×1.4NA objective, a Nikon Intensilight Mercury based light source and either Chroma #89014ET (three-color) or #89000ET (four-color) single excitation and emission filters (Chroma Technology Corp.) with the excitation and emission filters installed in Prior motorized filter wheels. A piezo electrically driven objective mount (PIFOC model P-721, Physik Instrumente) was used to control movement in the z dimension. Step size for z stacks was set at 0.2 µm. Nikon Nis-Elements software was used to perform hardware control, image capture, and analysis. Images were deconvolved using a calculated point spread function with the constrained iterative algorithm of Volocity (PerkinElmer). The quantitation module of Volocity was used to calculate interprobe distances. To eliminate the possibility of measuring sister chromatids, only alleles with single probe signals were analyzed.

### Generation of knockout mice

Mouse work was approved by the UCSF IACUC, protocol number AN181381, and was conducted in accordance with AALAC and NIH guidelines. The 12 kb acheiropodia-associated sequence (chr7:156,608,724-156,620,764; hg19) was converted to mouse sequence (chr5:29,335,354-29,348,393; mm10) using the UCSC Genome Broswer^36^ liftOver tool. Two gRNA were designed to target the 5′ and 3′ ends of this region (Supplementary Table 3) using the gRNA design tool on the Integrated DNA Technologies (IDT) website and selected based on low off-target and high on-target scores. The acheiropodia deletion allele was generated using *i*-GONAD^39^. Briefly, after reconstitution of two crRNA (IDT) and tracrRNA (IDT), these were mixed together (final concentration 100 µM each) and incubated at 92 °C for 2 min and left at room temperature for 10 min to prepare the crRNA/tracrRNA complex. The genome-editing mixture, (30 µM crRNA/tracrRNA complex, 1 mg/ml Cas9 protein (IDT), Opti-MEM) was incubated at 37 °C for 10 min. Estrus female FVB mice (Jackson Laboratory, catalog no. 001800) were mated to male mice the night before. The presence of copulation plugs was confirmed by visual inspection the next morning and the females having plugs were designed as Day 0.5 of gestation at noon and Day 0.7 of gestation at 16:00. Females on Day 0.7 were used for oviduct electroporation. Mice were anesthetized using isoflurane, the ovary and oviducts were exposed by grasping the adipose tissue surrounding the ovary. Approximately 1-2 μl of the genome-editing mixture was injected into the oviduct lumen upstream of the ampulla using a micropipette. Immediately following injection, the oviduct was covered with a piece of wet paper soaked in PBS and then grasped by tweezer-type electrodes (Bulldog Bio, catalog no. CUY652P2.5 ×4). The electroporation was performed using a square-wave pulse generator BTXECM830 (BTX Genetronics Inc.). The electroporation conditions used were 8 pulses of 50 V at 5 mseconds wavelength. After electroporation, the oviducts were placed in their original position, and the muscle layer incision was sutured using absorbable suture chromic gut. The coat layer incision was closed by AutoClip kit (Fine Science Tools, catalog no. 12022-09). The animals were kept on a warming pad (37 °C) during surgery and monitored for anesthesia recovery following surgery.

### Sanger sequencing and Southern blot

PCR-Sanger sequencing (primers provided in Supplementary Table 3) was preformed using standard techniques^28^. For Southern blot analyses, genomic DNA were treated with *Bst*XI (New England Biolabs, catalog no. R0113) and fractionated by agarose gel electrophoreses. Following capillary transfer onto nylon membranes, blots were hybridized with Digoxigenin (DIG)-labeled DNA probes (corresponding to chr5:29,348,565-29,349,037; mm10) amplified by the PCR DIG Probe Synthesis Kit (Sigma-Aldrich, catalog no. 11636090910). The hybridized probe was immunodetected with anti-digoxigenin Fab fragments conjugated to alkaline phosphatase (Sigma-Aldrich, catalog no. 11093274910) and visualized with a CDP star (Sigma-Aldrich, catalog no. 11685627001) according to the manufacturer’s protocol. Chemiluminescence was detected using the FluorChem E (ProteinSimple, catalog no.92-14860-00).

### RT-qPCR

Total RNA was collected from E11.5 limb buds or lymphoblastoid cells using TRIzol (Thermo Fisher Scientific, catalog no. 15596026) and converted to cDNA using ReverTra Ace qPCR-RT master mix with genomic DNA (gDNA) remover (Toyobo, catalog no. FSQ-301). qPCR was performed using SsoFast EvaGreen supermix (Bio Rad, catalog no. 1725205). Primer sequences used for qPCR are shown in Supplementary Table 3.

### Whole-mount in situ hybridization

Mouse E11.5 embryos were fixed in 4% paraformaldehyde. A plasmid containing mouse *Shh* cDNA (GenScript, catalog no. OMu22903D) was used as template for DIG-labeled probes. Mouse whole-mount in situ hybridization was performed according to standard procedures^68^.

### Bone and cartilage staining

Embryos were harvested at E18.5 and limbs were dissected out and used for staining. Alcian blue/Alizarin red staining was performed according to standard procedures for late-gestation stage embryos^69^.

### Analysis of CTCF Hi-ChIP data

Analysis of the CTCF Hi-ChIP^35^ data and figure generation were done using the HiCExplorer^70^.

### Reporting summary

Further information on research design is available in the Nature Research Reporting Summary linked to this article.

## Supplementary information

Supplementary Information

Reporting Summary

## Data Availability

hg19 and hg38 human reference genome is available from NCBI (GenBank assembly “GCA_000001405.1”, and “GCA_000001405.15”, respectively). The deleted sequence information is available from Decipher database “#411659”. ChIP-seq and 4C-seq data are available from the Gene Expression Omnibus under accession number “GSE155324”. ENCODE data are available from the “UCSC genome browser [https://genome.ucsc.edu/]”. Hi-C datasets are available from the “3D Genome Browser [http://www.3dgenome.org/]”. The CTCF motif was obtained from “JASPAR [http://jaspar.genereg.net/]”. All other relevant data supporting the key findings of this study are available within the article and its Supplementary Information files or from the corresponding author upon reasonable request. A reporting summary for this article is available as a Supplementary Information file. Source data are provided with this paper.

## Source data

Source Data
