# Supplementary material for: Deletion of CTCF sites in the SHH locus alters enhancer–promoter interactions and leads to acheiropodia
Source: Nat Commun. 2021 Apr 16;12:2282. doi: 10.1038/s41467-021-22470-z (PMC8052326; doi:10.1038/s41467-021-22470-z)
Supplement: Supplementary file 1 — Supplementary Information [file 41467_2021_22470_MOESM1_ESM.pdf]

Supplementary information

Deletion of CTCF sites in the *SHH* locus alters enhancer-promoter interactions and leads to acheiropodia

Aki Ushiki[1,2], Yichi Zhang[1,2,3], Chenling Xiong[1,2], Jingjing Zhao[1,2], Ilias Georgakopoulos-Soares[1,2], Lauren Kane[4], Kirsty Jamieson[2,5], Michael J. Bamshad[6,7,8], Deborah A. Nickerson[7,8], University of Washington Center for Mendelian Genomics, Yin Shen[2,5], Laura A. Lettice[4], Elizabeth Lemos Silveira-Lucas[9], Florence Petit[10], Nadav Ahituv[1,2*]

[1] Department of Bioengineering and Therapeutic Sciences, University of California San Francisco, San Francisco, CA 94158, USA.

[2] Institute for Human Genetics, University of California San Francisco, San Francisco, CA 94158, USA.

[3] School of Pharmaceutical Sciences, Tsinghua University, Beijing, 100084, China

[4] MRC Human Genetics Unit, Institute of Genetics and Molecular Medicine, University of Edinburgh, Crewe Road, Edinburgh EH4 2XU, UK

[5] Department of Neurology, University of California San Francisco, San Francisco, CA 94158, USA.

[6] Department of Pediatrics, University of Washington, Seattle, WA 98195, USA.

[7] Department of Genome Sciences, University of Washington, Seattle, WA 98195, USA.

[8] Brotman-Baty Institute, Seattle, WA 98195, USA.

[9] Consultorio Genetica Clinica, Porto Alegre, 90440, Brazil

[10] CHU Lille, University of Lille, EA7364 RADEME, F-59000, Lille, France.

Supplementary information for UW-CMG

University of Washington Center for Mendelian Genomics (UW-CMG)

Peter Anderson[11], Suzanne M. Leal[12], Tamara J. Bacus[11], Elizabeth E. Blue[11], Kati J. Buckingham[11], Jessica X. Chong[11], Diana Cornejo Sánchez[12], Colleen P. Davis[11], Christian D. Frazar[11], Danielle Giroux[11], William W. Gordon[11], Martha Horike-Pyne[11], Jameson R. Hurless[11], Gail P. Jarvik[11], Eric Johanson[11], J. Thomas Kolar[11], Melissa P. MacMillan[11], Colby T. Marvin[11], Sean McGee[11], Daniel J. McGoldrick[11], Betselote Mekonnen[11], Patrick M. Nielsen[11], Karynne Patterson[11], Benjamin Pelle[11], Aparna Radhakrishnan[11], Matthew A. Richardson[11], Gwendolin T. Roote[11], Erica L. Ryke[11], Isabelle Schrauwen[12], Kathryn M. Shively[11], Joshua D. Smith[11], Monica Tackett[11], Machiko S. Threlkeld[11], Gao Wang[12], Jeffrey M. Weiss[11], Marsha M. Wheeler[11], Qian Yi[11], Jordan E. Zeiger[11], and Xiaohong Zhang[11]

[11] University of Washington

[12] Columbia University

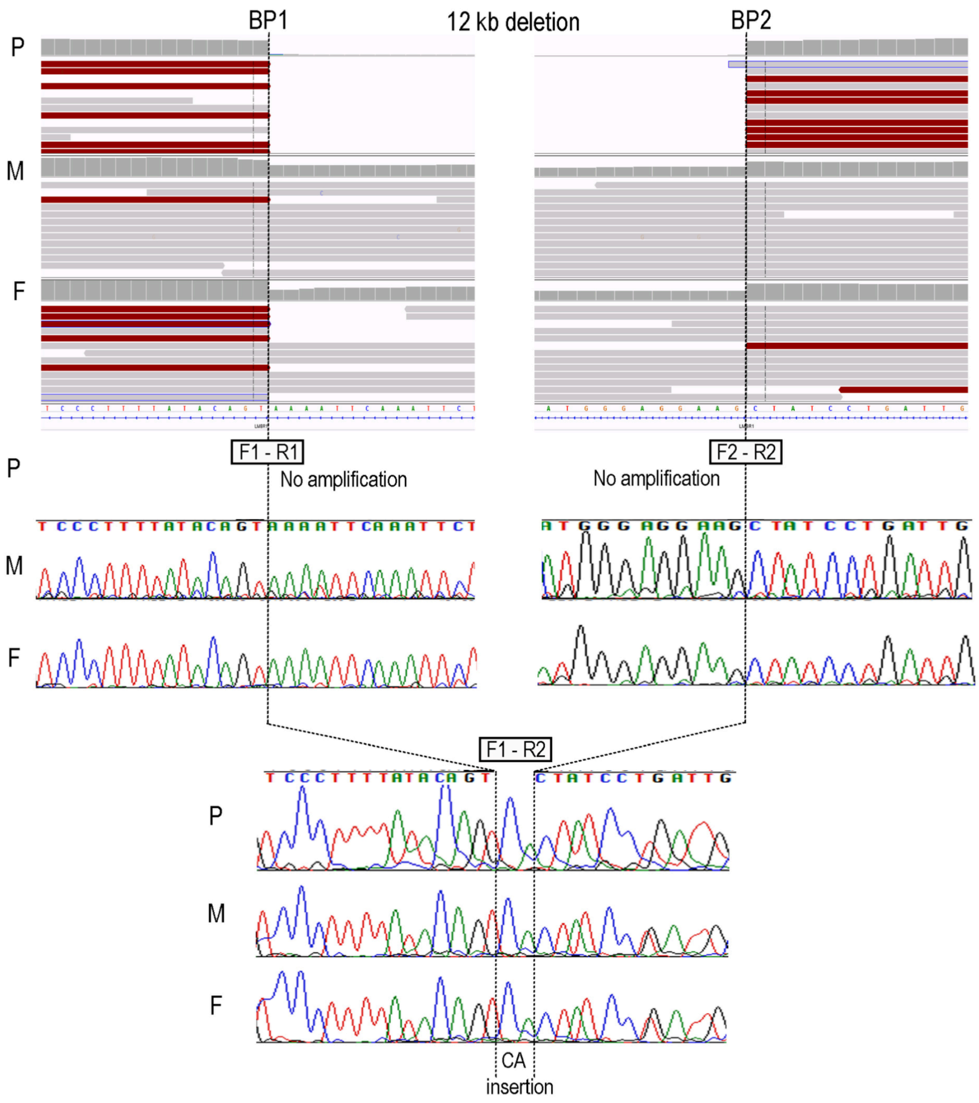

Supplementary Fig. 1 | Sequencing analyses of the acheiropodia-associated deletion.

WGS alignments showing the homozygous 12 kb deletion in the acheiropodia proband, with red lines indicating truncated reads (upper panel). The deletion appears heterozygous in both parents. Sanger sequencing of the breakpoint in the acheiropodia proband and her parents using three different primer pairs (lower). Variant Chr7(GRCh37):g.156608724_156620764delinsCA is homozygous in the proband and heterozygous in the parents. BP: breakpoint; P: proband; M: mother; F: father.

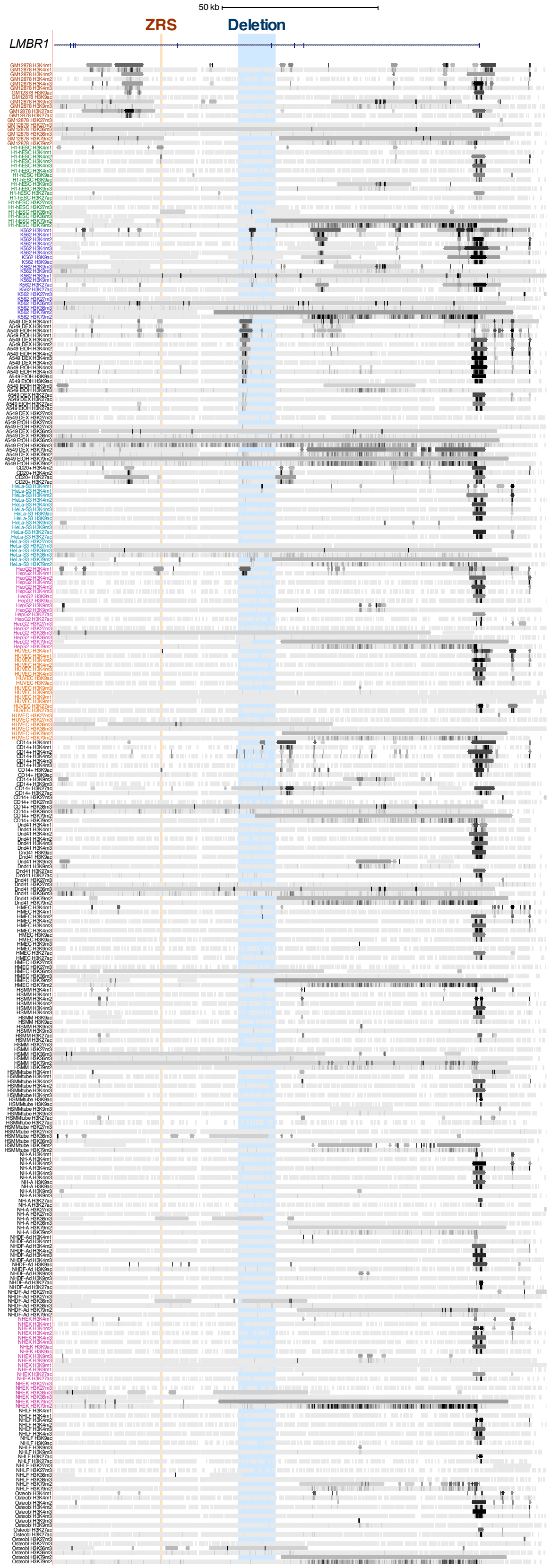
50 kb
ZRS
Deletion
LMBR1

Supplementary Fig. 2 | ENCODE histone modification ChIP-seq peaks in the acheiropodia-associated deleted region.

ChIP-seq datasets of ten different histone modifications from 18 different cell lines around the 12 kb acheiropodia-associated deletion (highlighted in blue).

Acheiropodia 12 kb deletion

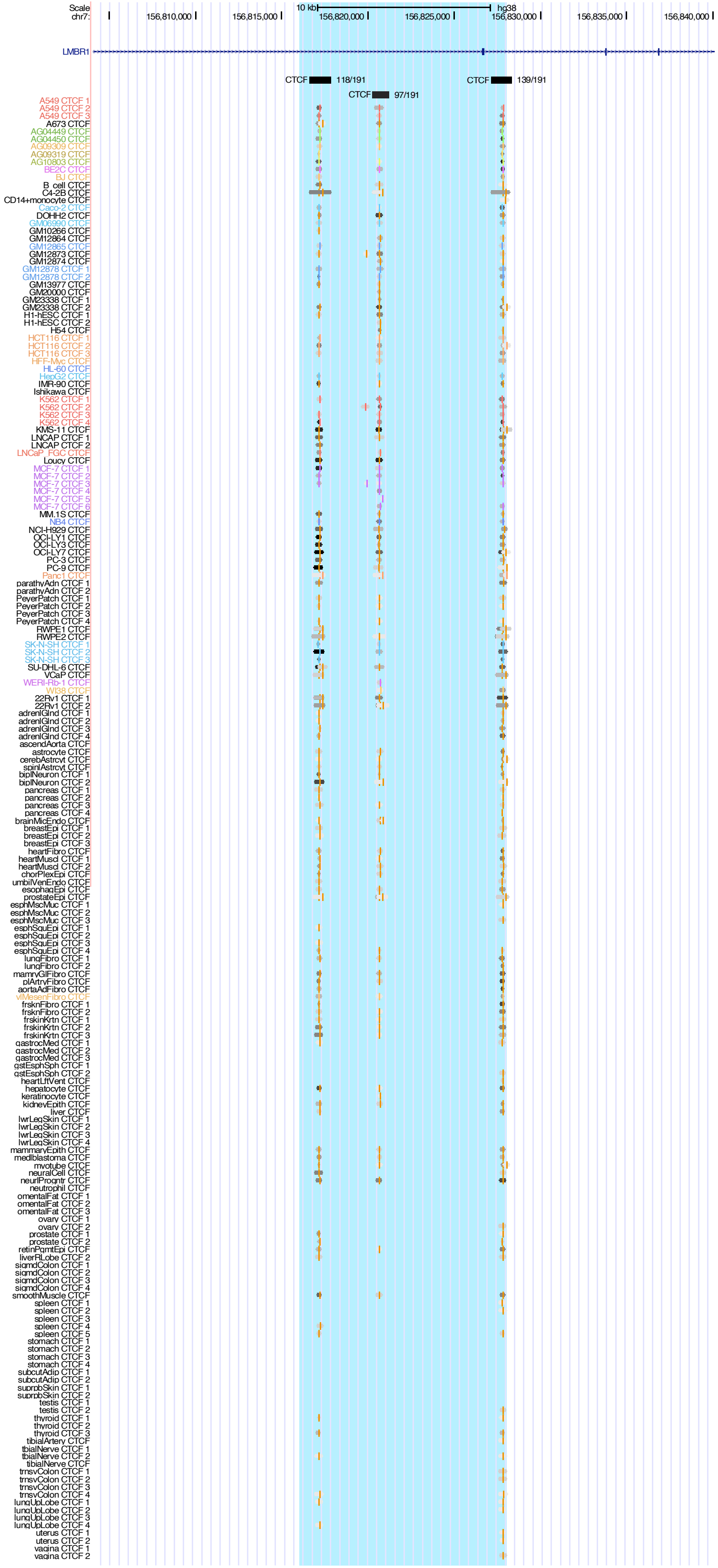

Supplementary Fig. 3 | ENCODE CTCF ChIP-seq peaks in the acheiropodia-associated deleted region.

CTCF ChIP-seq peaks around the 12 kb acheiropodia-associated deletion (highlighted in blue) showing the three CTCF sites located in this region. The number of ChIP-seq assays that have a significantly enriched CTCF peak versus the number of assays are noted for each peak in the track above and below are all the individual assay results.

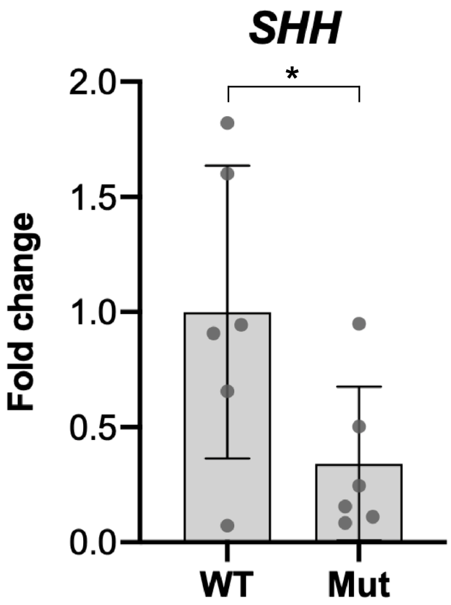

Supplementary Fig. 4 | *SHH* gene expression levels in the lymphoblastoid cell lines.

SHH gene expression levels from wild type (WT) and acheiropodia (Mut) lymphoblastoid cells as determined by qRT-PCR. Each value represents the ratio of *SHH* gene expression to that of *hypoxanthine phosphoribosyltransferase 1* gene, and values are mean ± standard deviation. The expression value of WT was arbitrarily set at 1.0. Each dot represents an individual replicate and statistical differences were determined using a two-sided unpaired *t*-test. Source data are provided as a Source Data file. **P*-value=0.0486.

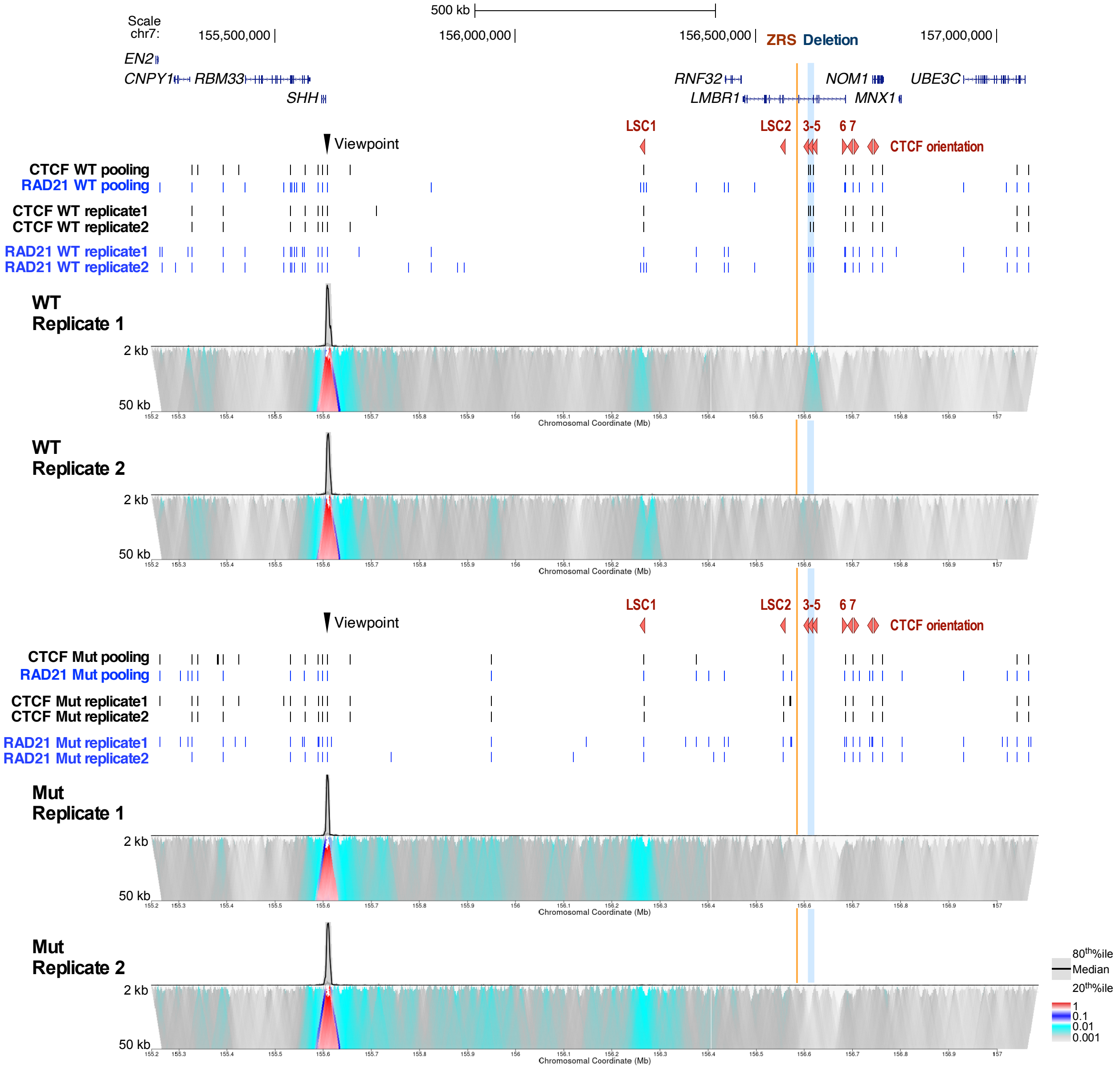

Supplementary Fig. 5 | ChIP-seq and 4C-seq replicate data in the *LMBR1-SHH* locus.

CTCF (black) and RAD21 (blue) ChIP-seq enrichment in lymphoblastoid cells from wildtype (WT) and proband (Mut) at the *LMBR1-SHH* locus for two technical replicates. The ZRS and the acheiropodia-associated deleted region are shown in orange and blue vertical lines respectively and CTCF orientations are shown as red triangles. Below are the results from two technical replicates of 4C-seq, showing 4C contact profiles in lymphoblastoid cells from wildtype (WT) and proband (Mut). The viewpoint is depicted

by a black arrowhead. The median and 20th and 80th percentiles of sliding 2-50 kb windows determine the main trend line. The color scale represents enrichment relative to the maximum medium value attainable at 12 kb resolution.

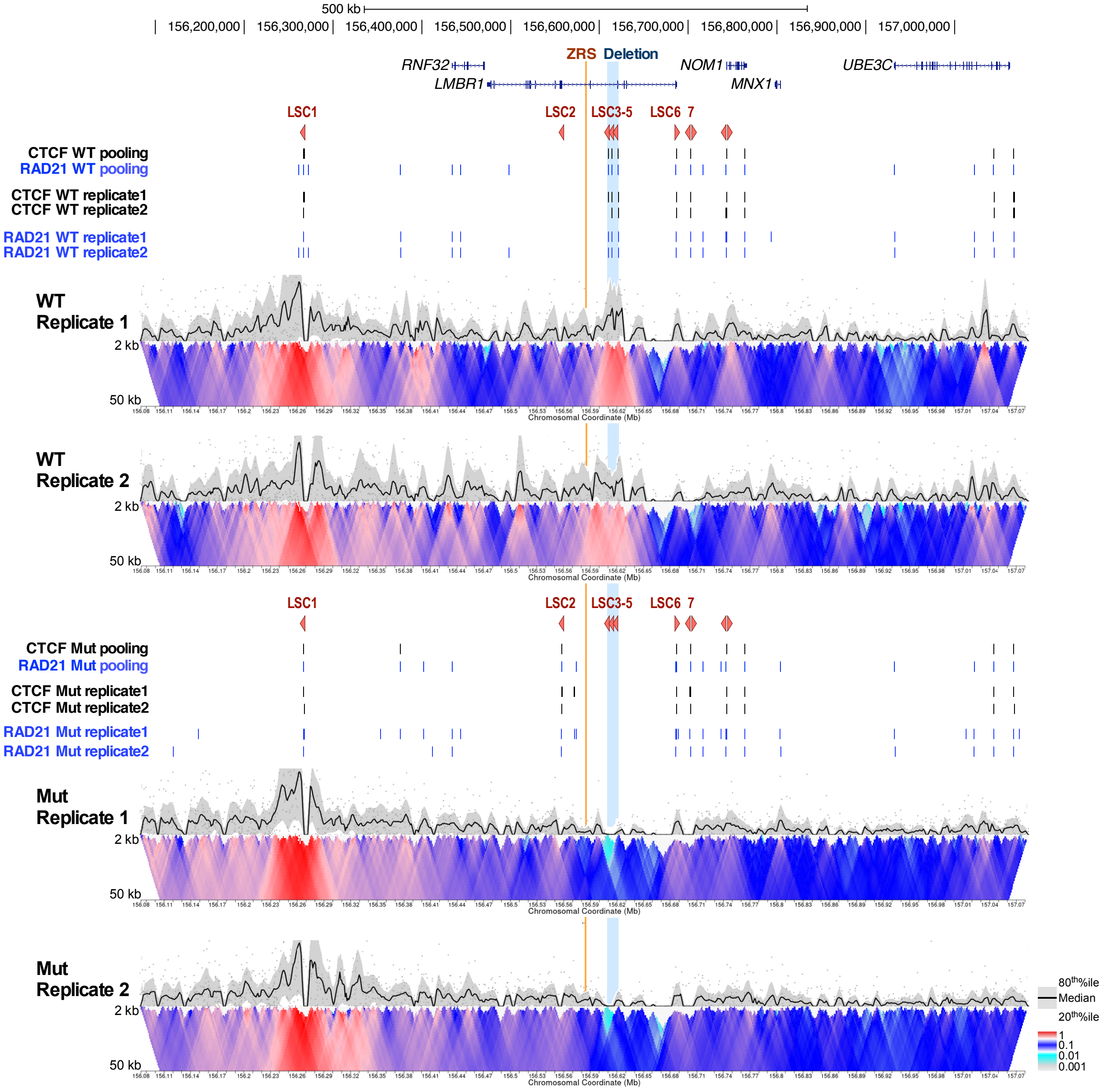

Supplementary Fig. 6 | ChIP-seq and 4C-seq replicate data around *LMBR1*.
CTCF (black) and RAD21 (blue) ChIP-seq enrichment in lymphoblastoid cells from wildtype (WT) and proband (Mut) at the *LMBR1* locus for two technical replicates. The ZRS and the acheiropodia-associated deleted region are shown in orange and blue vertical lines respectively and CTCF orientations are shown as red triangles. Below are the results from two technical replicates of 4C-seq, showing 4C contact profiles in lymphoblastoid cells from wildtype (WT) and proband (Mut). The viewpoint is depicted

by a black arrowhead. The median and 20th and 80th percentiles of sliding 2-50 kb windows determine the main trend line. The color scale represents enrichment relative to the maximum medium value attainable at 12 kb resolution.

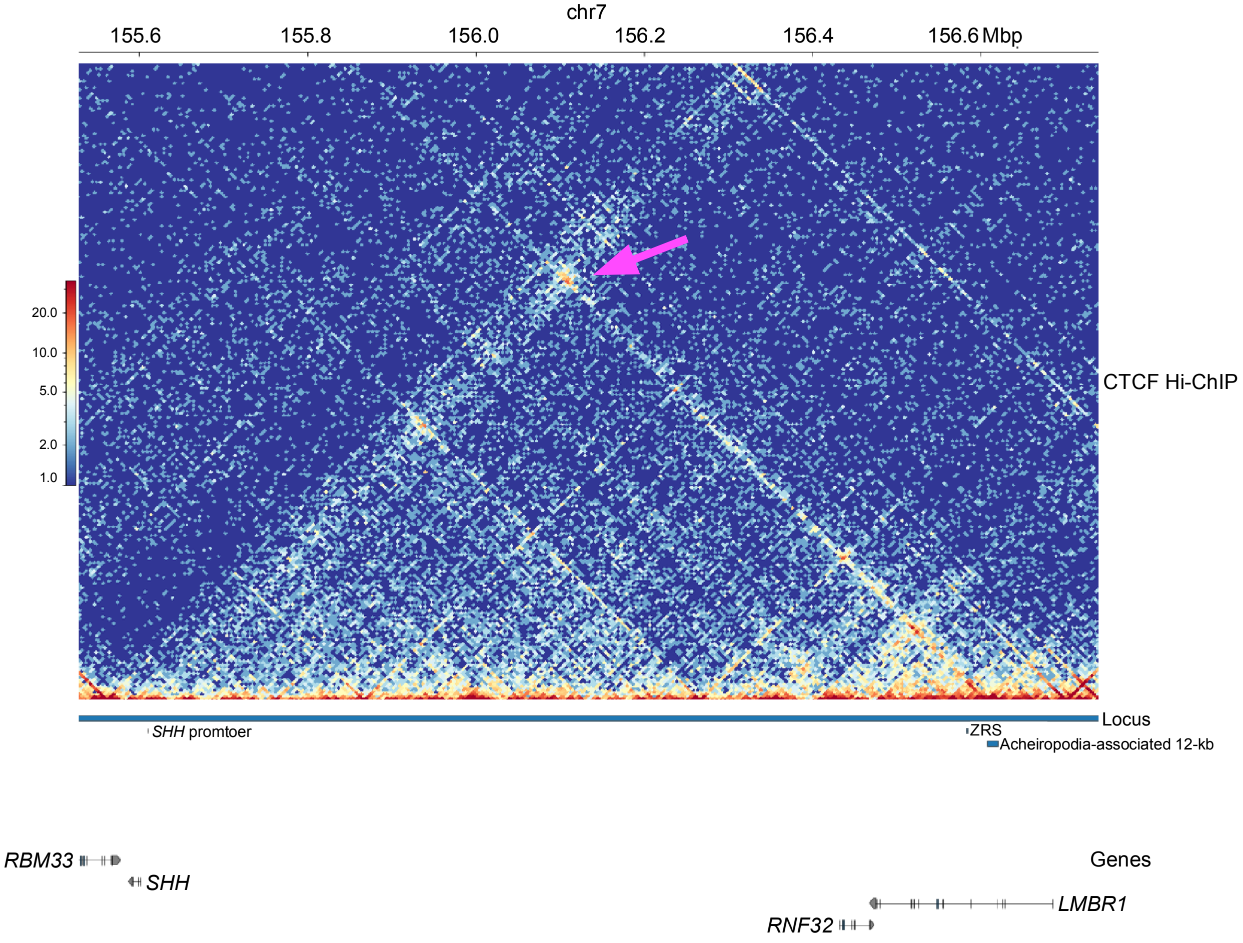

Supplementary Fig. 7 | CTCF Hi-ChIP Interactions in GM12878 cell line.
Interactions in the *SHH* locus from CTCF Hi-ChIP data[1]. The pink arrow indicates the interaction between the *SHH* promoter and the acheiropodia associated 12 kb region.

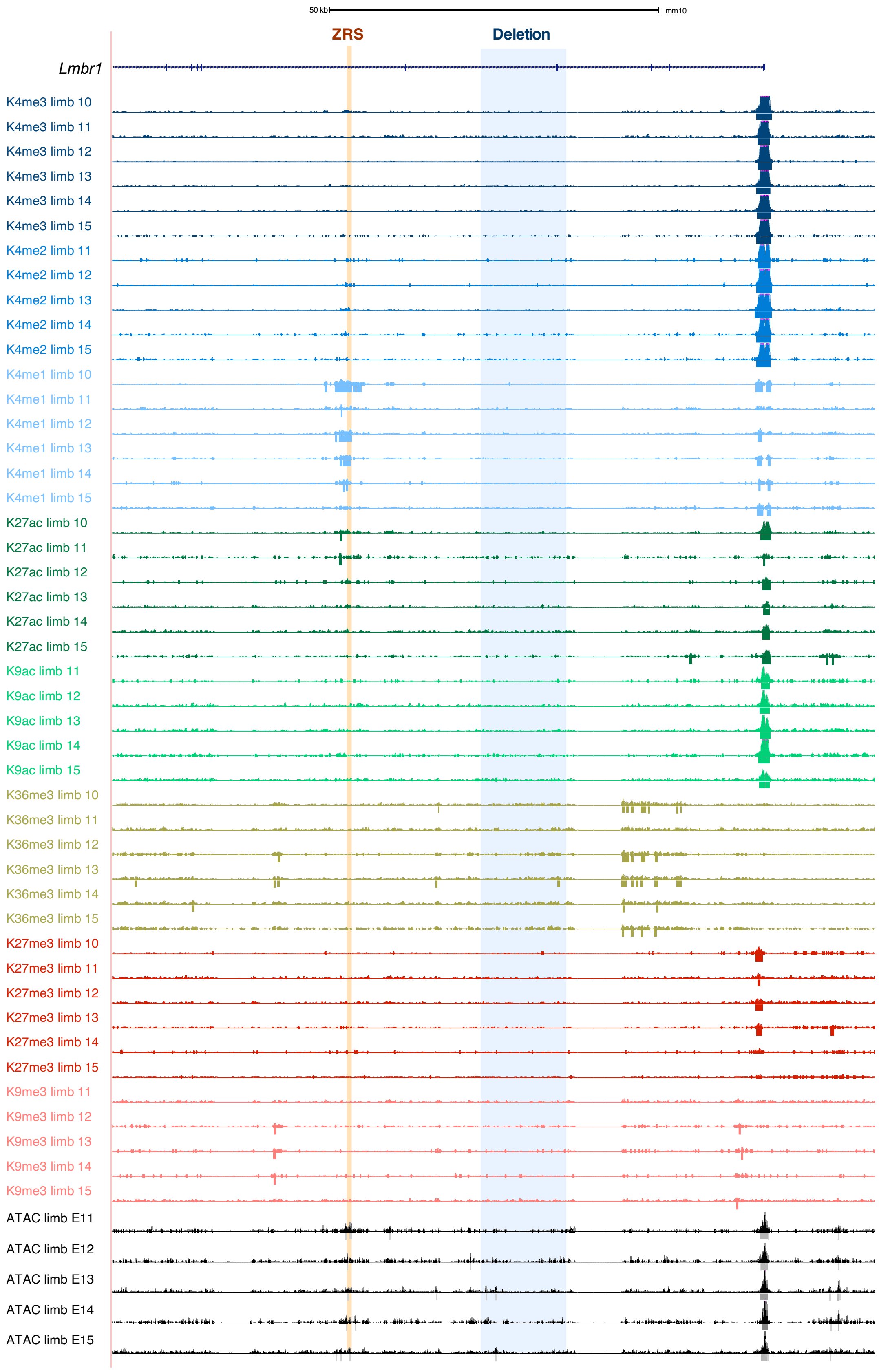

50 kb
mm10
ZRS
Deletion
Lmbr1
K4me3 limb 10
K4me3 limb 11
K4me3 limb 12
K4me3 limb 13
K4me3 limb 14
K4me3 limb 15
K4me2 limb 11
K4me2 limb 12
K4me2 limb 13
K4me2 limb 14
K4me2 limb 15
K4me1 limb 10
K4me1 limb 11
K4me1 limb 12
K4me1 limb 13
K4me1 limb 14
K4me1 limb 15
K27ac limb 10
K27ac limb 11
K27ac limb 12
K27ac limb 13
K27ac limb 14
K27ac limb 15
K9ac limb 11
K9ac limb 12
K9ac limb 13
K9ac limb 14
K9ac limb 15
K36me3 limb 10
K36me3 limb 11
K36me3 limb 12
K36me3 limb 13
K36me3 limb 14
K36me3 limb 15
K27me3 limb 10
K27me3 limb 11
K27me3 limb 12
K27me3 limb 13
K27me3 limb 14
K27me3 limb 15
K9me3 limb 11
K9me3 limb 12
K9me3 limb 13
K9me3 limb 14
K9me3 limb 15
ATAC limb E11
ATAC limb E12
ATAC limb E13
ATAC limb E14
ATAC limb E15

Supplementary Fig. 8 | Mouse ENCODE histone modification ChIP-seq and ATAC-seq peaks around *Lmbr1*.

Histone modification (H3K4me3, H3K4me1, H3K27ac, H3K9ac, H3K36me3, H3K27me3, H3K9me3) ChIP-seq and ATAC-seq peaks from mouse E10.5-E15.5 limbs. The ZRS and the acheiropodia-associated deleted region are shown as orange and blue vertical lines respectively.

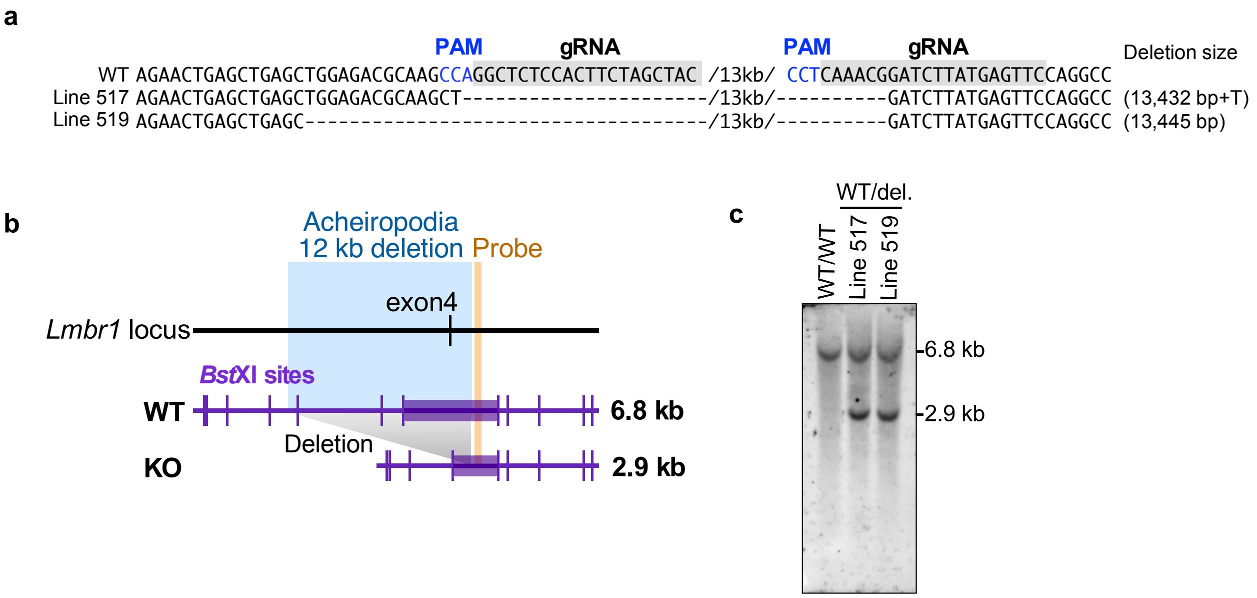

Supplementary Fig. 9 | Sanger sequencing and southern blot analyses of the 12 kb mouse deleted region.

a, Sanger sequencing results for mouse line 517 and 519. The PAM motif is depicted in blue font and gRNA sequences are shaded in gray. **b**, Location of the *Bst*XI restriction enzyme sites (purple lines) in wild type (WT) and knockout (KO) loci. The deletion and probe location are shown as blue and orange rectangle respectively. Expected band sizes in WT and KO are written to the right of the map. **c**, Southern blot analyses of wild type (WT) and heterozygous knockout mice with the estimated band size written to the right of the blot. Southern blot analyses were performed several times using founder and F1 generation.

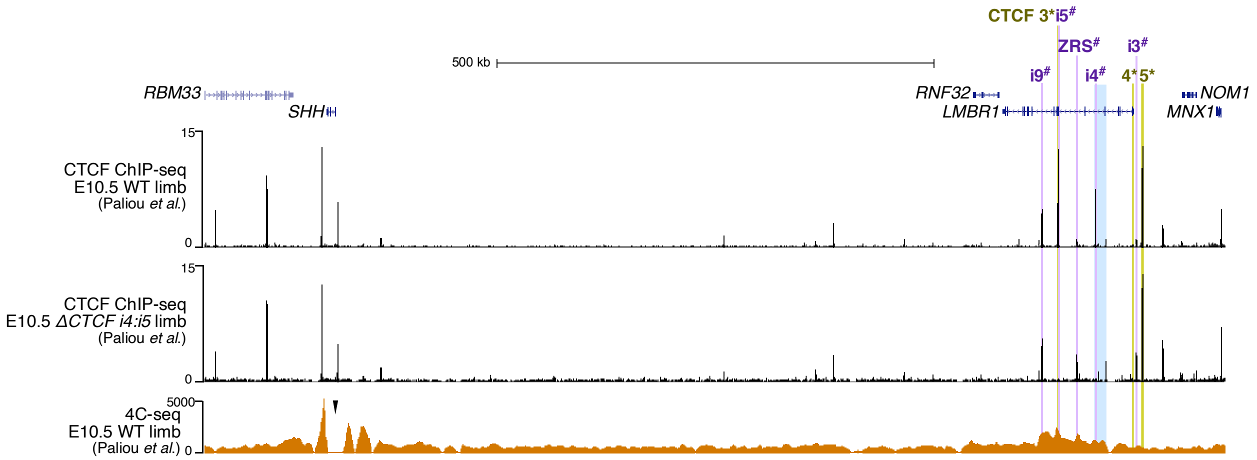

Supplementary Fig. 10 | Mouse CTCF ChIP-seq and 4C-seq peaks around the *Lmbr1-Shh locus*.

Mouse E10.5 limb CTCF ChIP-seq data and 4C-seq data from Paliou *et al.*[2]. The *Shh* viewpoint is depicted via a black arrowhead. CTCF site deletions analyzed by Paliou *et al*.[2] are marked by purple lines and # and those generated by Williamson *et al*.[3] are denoted by yellow lines or gray rectangle, and marked by *.

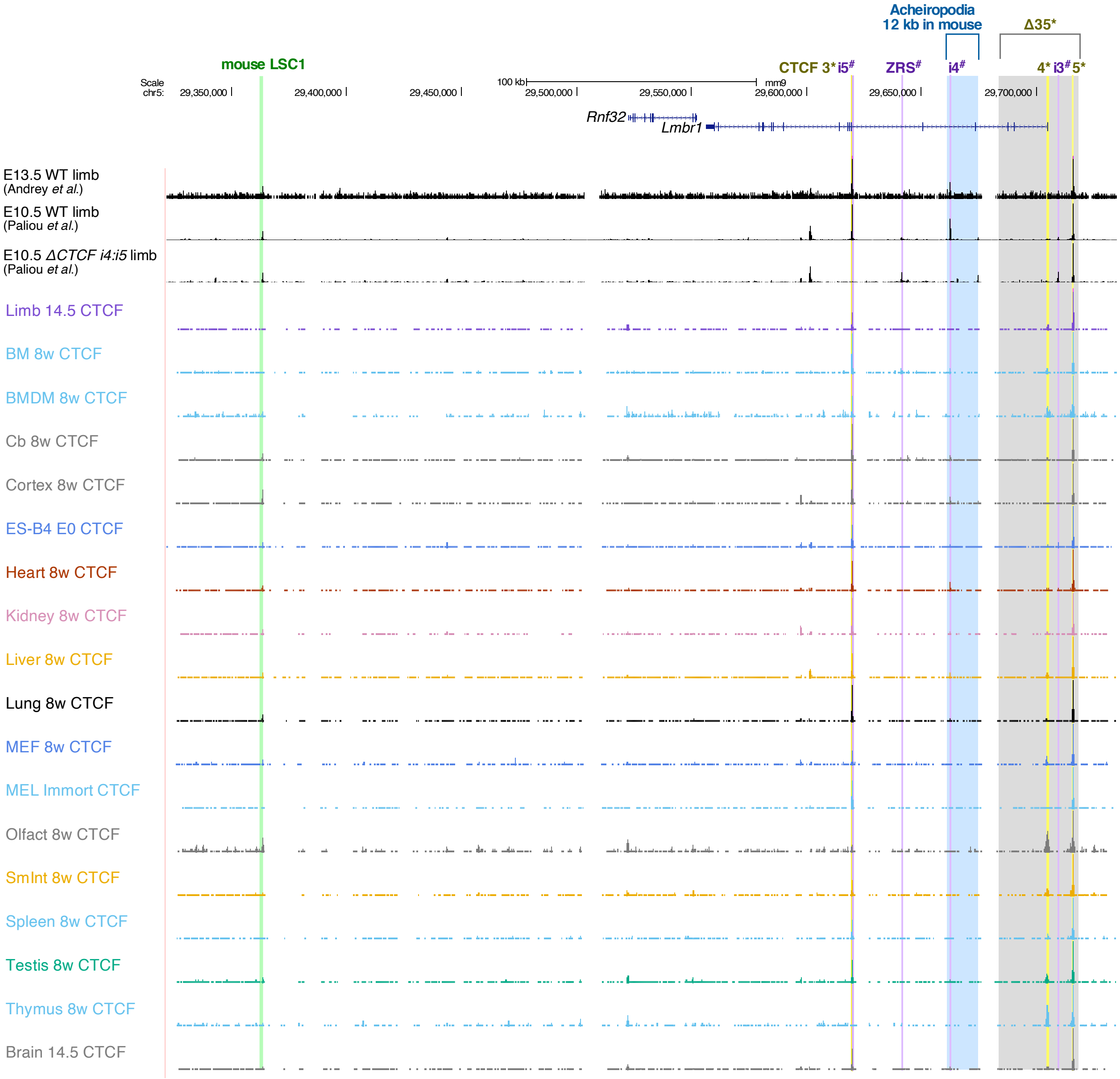

Supplementary Fig. 11 | Mouse ENCODE CTCF ChIP-seq peaks around *Lmbr1*. CTCF ChIP-seq from developing mouse limbs[3,2] and ENCODE[4] from various tissues/cells. CTCF site deletions analyzed by Paliou *et al.*[2] are marked by # and purple lines and those generated by Williamson *et al.*[3] are denoted by yellow lines or gray rectangle and marked by *. The mouse LSC1 CTCF site is marked by a green line. The mouse orthologous sequence for the acheiropodia-associated deletion is depicted by a blue rectangle.

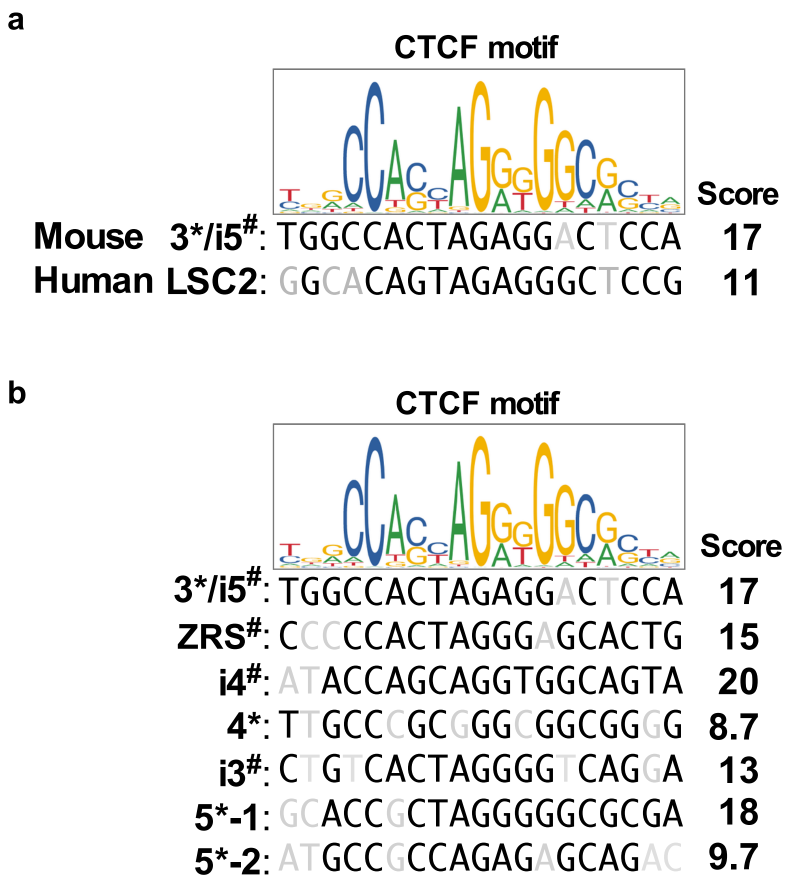

Supplementary Fig. 12 | Mouse CTCF motifs around the *Lmbr1* gene.

a-b, CTCF motif from JASPAR[5] [http://jaspar.genereg.net/] and motif scores, as assigned by FIMO[6], for 3*/i5# mouse and human LSC2 (**a**) and mouse CTCF sequences analyzed in previous studies[3,2] (**b**). The 5* contains two CTCF sites and shown as 5*-1 and 2.

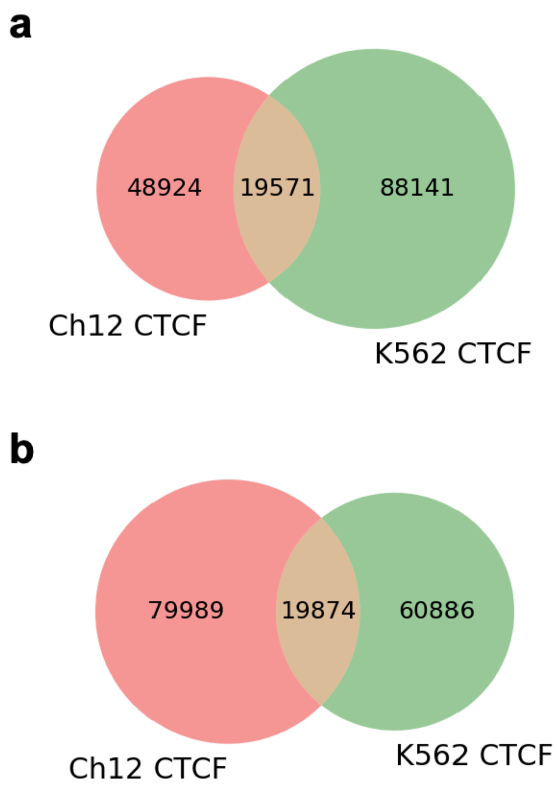

Supplementary Fig. 13 | Venn diagram showing overlap between human and mouse CTCF ChIP-seq peaks.

a, Venn diagram showing the overlap between CH12 CTCF-ChIP peaks that were converted to human (hg19) using the liftOver tool in the UCSC Genome Browser[7] with human K562 CTCF-ChIP peaks. **b**, Venn diagram showing the overlap between K562 CTCF-ChIP peaks that were converted to mouse (mm9) using the liftOver tool in the UCSC Genome Browser[7] with mouse CH12 CTCF-ChIP peaks.

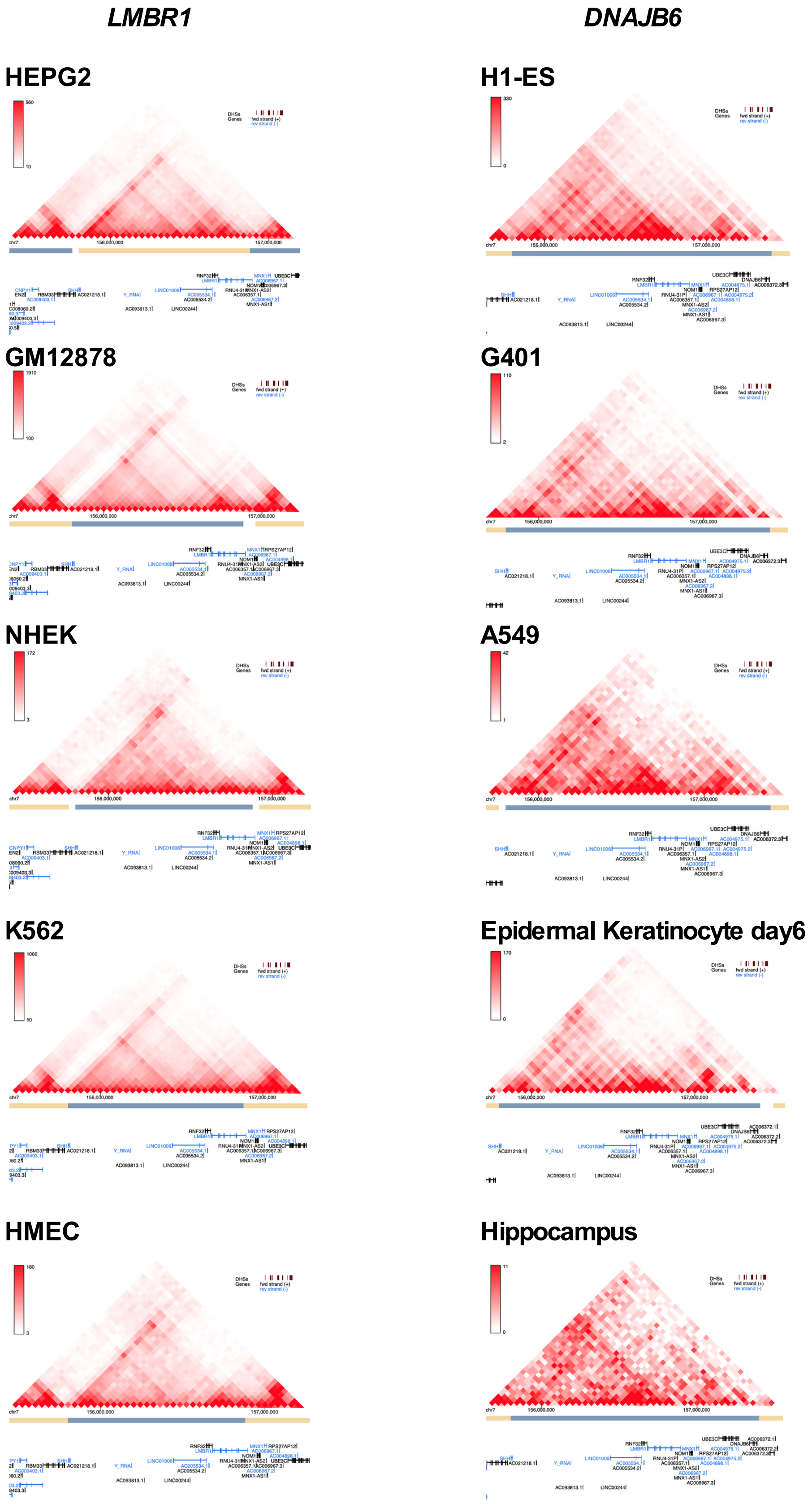
LMBR1
DNAJB6
HEPG2
H1-ES
GM12878
G401
NHEK
A549
K562
Epidermal Keratinocyte day6
HMEC
Hippocampus

Supplementary Fig. 14 | TAD boundaries in the human *LMBR1-SHH* locus from ten different cell types.

Hi-C datasets analyzed via the 3D Genome Browser[8] for ten different human cells lines showing the location of the TAD boundaries in the *SHH-LMBR1* locus. Above each Hi-C heatmap is the name of the cell type/tissue, gray and orange bars show the TAD structure and below are the location of the various genes in this locus.

Supplementary Table 1 | Homozygosity mapping results for individual VI-2, affected with acheiropodia.

CHR	SNP1	SNP2	POS1	POS2	KB	NSNP	DENSITY	PHOM	PHET
1	rs2744778	rs537951	26013252	27374218	1360.966	538	2.53	0.998	0.002
1	rs10437318	rs272817	35403677	36646491	1242.814	302	4.115	1	0
1	rs7556311	exm58332	49123567	51584465	2460.898	150	16.406	0.993	0.007
1	exm92700	exm95637	147380101	150991132	3611.031	439	8.226	0.991	0.009
1	rs3131323	rs3908502	177719196	180591237	2872.041	631	4.552	0.998	0.002
2	exm201593	exm2255484	72359400	73466812	1107.412	128	8.652	0.984	0.016
2	kgp8786363	exm216367	99554388	100623395	1069.007	198	5.399	0.995	0.005
2	exm260784	exm2254932	209138368	210810644	1672.276	230	7.271	0.987	0.013
3	rs722615	rs2346021	5899967	11700713	5800.746	1360	4.265	0.996	0.001
3	exm319359	exm323508	51968725	52988425	1019.7	727	1.403	0.997	0.003
3	rs11128158	rs1437528	66668365	77950703	11282.338	1552	7.27	0.999	0
3	rs9861237	rs6800203	85402469	86466286	1063.817	103	10.328	0.981	0.019
3	rs1838554	rs16826629	105927328	117017073	11089.745	2040	5.436	1	0
3	rs990739	rs11919380	142926765	148993158	6066.393	842	7.205	1	0
3	rs4856678	rs13082820	162092216	164926605	2834.389	285	9.945	0.996	0.004
3	rs6778567	rs12489012	192611340	197833758	5222.418	1286	4.061	0.998	0
4	rs7693293	rs13152405	5093523	14497339	9403.816	1995	4.714	0.997	0.001
4	rs7693369	exm431870	158778839	159894391	1115.552	191	5.841	0.99	0.01
5	exm-rs9292862	rs4866772	43028221	44208106	1179.885	143	8.251	0.993	0.007
5	rs1011814	rs7293455	44335820	46399093	2063.273	107	19.283	0.991	0.009
5	rs6888236	exm476641	130215911	131649405	1433.494	197	7.277	0.99	0.01
5	rs437220	exm2261872	160545841	164306736	3760.895	418	8.997	0.998	0.002
6	exm2270422	exm584615	145677803	146755072	1077.269	127	8.482	0.984	0.016
7	rs6956395	rs1425132	28072245	37562368	9490.123	1642	5.78	0.999	0.001
7	rs17139634	newrs148500031	64902831	66458309	1555.478	105	14.814	1	0
**7**	**rs12719966**	**rs1985369**	**155149037**	**159119220**	**3970.183**	**715**	**5.553**	**0.999**	**0.001**
8	exm687695	rs10958798	22031177	43791691	21760.514	4002	5.437	0.999	0
8	rs7836486	rs10111120	46942842	51564759	4621.917	383	12.068	0.997	0.003
8	rs1560846	rs4739127	77529466	78749262	1219.796	140	8.713	1	0
8	exm711859	indel.101907	99168279	101020788	1852.509	265	6.991	0.989	0.011
8	rs10093778	exm716475	112488761	113563079	1074.318	111	9.679	0.991	0.009
9	rs7023466	rs10046802	93005808	94437294	1431.486	201	7.122	0.995	0.005
9	exm772873	rs3750473	115336625	121468282	6131.657	1426	4.3	0.999	0.001
11	exm869284	rs7933771	193146	3851383	3658.237	1720	2.127	0.999	0.001
11	rs747190	exm2271460	8360804	9383450	1022.646	264	3.874	0.992	0.004
11	rs1228020	rs7944067	47953477	51525878	3572.401	340	10.507	0.991	0.009
11	rs34944111	exm909841	54794237	56310621	1516.384	639	2.373	0.997	0.002
11	rs6590849	rs2509689	101218094	102368719	1150.625	202	5.696	0.99	0.01
11	exm959555	exm963044	118037610	119290776	1253.166	671	1.868	1	0
13	rs861014	rs9538147	30629904	59200490	28570.586	4734	6.035	0.999	0
13	rs9542637	rs9516998	71849406	98318333	26468.927	3392	7.803	0.999	0
13	exm2267622	rs7991842	105386176	111056745	5670.569	999	5.676	0.999	0
14	rs10132552	rs10149476	101301012	107287663	5986.651	1201	4.985	0.999	0.001
15	exm1156252	exm1157672	43767774	44918698	1150.924	284	4.053	0.996	0.004
15	rs12594225	rs2163369	56102536	57164889	1062.353	171	6.213	0.982	0.012
15	rs4369593	rs736827	59697016	79358866	19661.85	4248	4.628	0.999	0
15	exm1182904	rs1256424	82336141	83524257	1188.116	174	6.828	0.983	0.017
16	exm2274844	rs11075685	66511561	68625435	2113.874	907	2.331	0.999	0.001
16	rs9783747	exm1255596	69458193	71319312	1861.119	432	4.308	0.988	0.009
16	rs9319574	rs2244455	82843167	84910657	2067.49	917	2.255	0.998	0.001
17	rs8082686	rs8067558	12545054	14598385	2053.331	410	5.008	0.998	0
17	exm1324419	exm1888089	40556776	41926141	1369.365	571	2.398	0.996	0.004
17	rs3921965	rs7209936	72031792	80926215	8894.423	3985	2.232	1	0
18	rs11875045	rs4798481	5193132	6693107	1499.975	241	6.224	1	0
18	rs4891065	rs12456851	73089473	78014582	4925.109	893	5.515	1	0
19	rs308054	exm1414040	3105987	6467966	3361.979	1291	2.604	0.998	0.001
19	rs10420999	rs2264683	22798253	24490299	1692.046	143	11.832	1	0
20	exm2260923	exm1533489	29419620	31619515	2199.895	419	5.25	0.99	0.007
20	rs6059703	newrs139021763	32768431	34055380	1286.949	384	3.351	0.997	0.003
20	exm1556774	exm1562040	61288242	62904863	1616.621	862	1.875	1	0
X	exm1625831	rs6527891	2700157	13686375	10986.218	1215	9.042	0.999	0.001
X	rs4333771	rs4313320	14167680	15424686	1257.006	119	10.563	0.992	0.008
X	rs2050064	rs5971539	29503266	31029701	1526.435	142	10.75	1	0
X	exm1637108	rs6520404	47917867	49049285	1131.418	208	5.44	0.99	0.01
X	rs4826654	exm1640749	50628606	53457578	2828.972	136	20.801	0.993	0.007
X	rs12841749	rs5965551	62620778	67555179	4934.401	268	18.412	1	0
X	rs28521091	indel.108442	76638703	77913836	1275.133	113	11.284	0.982	0.009
X	rs1841135	exm1656858	124669231	129058951	4389.72	322	13.633	0.997	0.003
X	exm2273223	rs11152537	181779	3582703	3400.924	150	22.673	1	0

In bold: homozygosity region comprising the *SHH-LMBR1* locus. CHR: Chromosome; SNP1: SNP at start of region; SNP2: SNP at end of region; POS1: Physical position (bp) of SNP1; POS2: Physical position (bp) of SNP2; KB: Length of region (kb); NSNP: Number of SNPs in run; DENSITY: Average SNP density (1 SNP per kb); PHOM: Proportion of sites homozygous; PHET: Proportion of sites heterozygous.

Supplementary Table 2 | Coordinates of human and mouse CTCF sites.

Coordinates for LSC1-7 and i3# are based on ChIP-seq peaks and i5#, ZRS#, i4# and CTCF3-5* are based on publications[3,2].

Name	Coordinate human (hg19)
LSC1	chr7:156266047-156266716
LSC2	chr7:156556580-156557319
LSC3	chr7:156609512-156610181
LSC4	chr7:156613060-156613729
LSC5	chr7:156620116-156620785
LSC6	chr7:156685860-156686529
LSC7	chr7:156701434-156702103

Name	Coordinate mouse (mm9)
LSC1	chr5:29363224-29363591
CTCF3*	chr5:29619575-29620326
CTCF4*	chr5:29704457-29705661
CTCF5*	chr5:29715776-29716437
i5#	chr5:29619921-29619969
ZRS#	chr5:29641336-29641385
i4#	chr5:29662545-29662612
i3#	chr5:29709136-29709762

Supplementary Table 3 |

Sequence of PCR primers, fosmid probes and sgRNAs used in this study.

Sanger sequence for acheiropodia family

Amplicon	Name	Sequence 5'>3'
LMBR1 wild-type allele	LMBR1-F1	ATGATGCAGGTTGTAACTACAG
	LMBR1-R1	GTGATTTGTGAGCATTGCCT
LMBR1 wild-type allele	LMBR1-F2	CTCAATAGACACCGCATTTCCT
	LMBR1-R2	TGGTGAGATTTTGGATTTGATCT
LMBR1 deleted allele	LMBR1-F1	ATGATGCAGGTTGTAACTACAG
	LMBR1-R2	TGGTGAGATTTTGGATTTGATCT
ZRS	ZRS-F1	GAGGTATAACCTCTGGCCAGTG
	ZRS-R1	CGCTTCCACCTGGTCAGTCC

Cloning for LacZ transgenic assay

5' and 3' end of 12 kb are in bold.
LacZ vector overlapping sequences for NEBuilder HiFi DNA Assembly cloning are italicized.

Amplicon	Name	Sequence 5'>3'
Acheiropodia associated 12 kb	LacZ-F	*GCTGCAGGAATTCGATATCA***AAAATTCAAATTCTTCTCTAGATGTCAAAGT**
	LacZ-R	*CGAGGTCGACGGTATCGATACGTA***CTTCCTCCCATGACTGGAGAAGC**

4C-seq

1st round PCR: Genomic regions are in bold.

2nd round PCR: P5 adapter (green), P7 adapter (blue), index (brown)

	Name	Sequence 5'>3'
1st round PCR for *SHH* promoter viewpoint	hSHH2-2step-4C-F	TACACGACGCTCTTCCGATCT**AGGAGCAGAGTTAAGCTGAGATC**
	hSHH2-2step-4C-R	ACTGGAGTTCAGACGTGTGCTCTTCCGATCT**TCCTCAATCCTCCGAGGCAC**
2nd round PCR for adopter/index	4C-2nd-Univ-F	AATGATACGGCGACCACCGAGATCTACACTCTTTCCCTACACGACGCTCTTCCGATCT
	4C-index2-R	CAAGCAGAAGACGGCATACGAGATACATCGGTGACTGGAGTTCAGACGTGTGCT
	4C-index23-R	CAAGCAGAAGACGGCATACGAGATATCCACTCGTGACTGGAGTTCAGACGTGTGCT
	4C-index5-R	CAAGCAGAAGACGGCATACGAGATCACTGTGTGACTGGAGTTCAGACGTGTGCT
	4C-index6-R	CAAGCAGAAGACGGCATACGAGATATTGGCGTGACTGGAGTTCAGACGTGTGCT
Hiseq4000 SE50 sequencing	Custom-reading-primer	CTTCCGATCTAGGAGCAGAGTTAAGCTGAGATC

gRNA for generating knockout mice

Name	PAM motif	Sequence 5'>3'
gRNA-1	TGG	GTAGCTAGAAGTGGAGAGCC
gRNA-2	AGG	GAACTCATAAGATCCGTTTG

Sanger sequencing for knockout mice

Amplicon	Name	Sequence 5'>3'
Mouse acheiropodia associated 12 kb	mLMBR12kb-KO/WT-F	GCATAGCCTAAGTGGCACAGGA
	mLMBR12kb-KO/WT-R	TGTGACTTGCTGACTTATCCCT

qPCR primer

Amplicon	Name	Sequence 5'>3'
Mouse *Shh* mRNA	ShhFw	ACCCCGACATCATATTTAAGGA
	ShhRev	TTAACTTGTCTTTGCACCTCTGA
Mouse *Beta-Actin* mRNA	Actb-F	GGCACCACACCTTCTACAATG
	Actb-R	GGGGTGTTGAAGGTCTCAAAC
Human *SHH* mRNA	hSHH-F2	GGAAGCAGCCTCCCGATTT
	hSHH-R2	AGTGGACATCACCACGTCTG
Human *HPRT* mRNA	hHPRT-F	TCCTTGGTCAGGCAGTATAATCC
	hHPRT-R	GTCAAGGGCATATCCTACAACAAA

Fosmid probes

Region	Fosmid ID	Coordinates (mm9)
SHH	G248P82324G11	Chr7: 155585515-155620891
LSC1	G248P83074E9	Chr7: 156248055-156288749
LSC2	G248P80007C10	Chr7: 156537478-156580173
LSC3-5	G248P8090C7	Chr7: 156591166-156630143

Supplementary References

1. Mumbach, M. R. *et al.* HiChIRP reveals RNA-associated chromosome conformation. *Nat. Methods* **16**, 489–492 (2019).
2. Paliou, C. *et al.* Preformed chromatin topology assists transcriptional robustness of Shh during limb development. *Proc. Natl. Acad. Sci. U. S. A.* **116**, 12390–12399 (2019).
3. Williamson, I. *et al.* Developmentally regulated Shh expression is robust to TAD perturbations. *Development* **146**, (2019).
4. Yue, F. *et al.* A comparative encyclopedia of DNA elements in the mouse genome. *Nature* **515**, 355–64 (2014).
5. Fornes, O. *et al.* JASPAR 2020: update of the open-access database of transcription factor binding profiles. *Nucleic Acids Res.* **48**, D87–D92 (2020).
6. Grant, C. E., Bailey, T. L. & Noble, W. S. FIMO: scanning for occurrences of a given motif. *Bioinformatics* **27**, 1017–8 (2011).
7. Kent, W. J. *et al.* The human genome browser at UCSC. *Genome Res.* **12**, 996–1006 (2002).
8. Wang, Y. *et al.* The 3D Genome Browser: a web-based browser for visualizing 3D genome organization and long-range chromatin interactions. *Genome Biol.* **19**, 151 (2018).
