# Supplementary material for: Deletion of CTCF sites in the SHH locus alters enhancer–promoter interactions and leads to acheiropodia
Source: Nat Commun. 2021 Apr 16;12:2282. doi: 10.1038/s41467-021-22470-z (PMC8052326; doi:10.1038/s41467-021-22470-z)
Supplement: Supplementary file 2 — Reporting Summary [file 41467_2021_22470_MOESM2_ESM.pdf]

nature research

Corresponding author(s): Nadav Ahituv

Last updated by author(s): Jan 13, 2021

Reporting Summary

Nature Research wishes to improve the reproducibility of the work that we publish. This form provides structure for consistency and transparency in reporting. For further information on Nature Research policies, see our Editorial Policies and the Editorial Policy Checklist.

Statistics

For all statistical analyses, confirm that the following items are present in the figure legend, table legend, main text, or Methods section.

n/a	Confirmed	
☐	☒	The exact sample size (n) for each experimental group/condition, given as a discrete number and unit of measurement
☐	☒	A statement on whether measurements were taken from distinct samples or whether the same sample was measured repeatedly
☐	☒	The statistical test(s) used AND whether they are one- or two-sided<br>*Only common tests should be described solely by name; describe more complex techniques in the Methods section.*
☒	☐	A description of all covariates tested
☒	☐	A description of any assumptions or corrections, such as tests of normality and adjustment for multiple comparisons
☐	☒	A full description of the statistical parameters including central tendency (e.g. means) or other basic estimates (e.g. regression coefficient) AND variation (e.g. standard deviation) or associated estimates of uncertainty (e.g. confidence intervals)
☐	☒	For null hypothesis testing, the test statistic (e.g. F, t, r) with confidence intervals, effect sizes, degrees of freedom and P value noted<br>*Give P values as exact values whenever suitable.*
☒	☐	For Bayesian analysis, information on the choice of priors and Markov chain Monte Carlo settings
☒	☐	For hierarchical and complex designs, identification of the appropriate level for tests and full reporting of outcomes
☒	☐	Estimates of effect sizes (e.g. Cohen's d, Pearson's r), indicating how they were calculated

Our web collection on statistics for biologists contains articles on many of the points above.

Software and code

Policy information about availability of computer code

Data collection	No software was used for data collection.
Data analysis	All ChIP-seq samples were processed according to ENCODE guidelines for transcription factor ChIP-seq analysis. Raw reads were aligned against GRCh37(hg19) using bowtie2 (2.2.6). Duplicate reads were marked using Picard(1.126)'s MarkDuplicates and multimapping , low quality, duplicated and non-properly paired reads were removed. Library complexity measures and flagstats were generated for each BAM file.<br>BAM files were converted to tagAlign format and two subsampled pseudoreplicates were generated for each sample with half the total reads. Peak calling, fold change and p-value signal tracks were generated using MACS2(v2.1.1). Irreproducible Discovery Rate (IDR) analysis was performed using self-pseudoreplicates and the main samples to obtain self-consistent sets of peaks. Final peak calls were filtered by ENCODE blacklist (Amemiya et al., 2019) and an IDR of 2% and a signal value > 30.<br>4C-seq raw reads were trimmed to 50 bp with cutadapt 2.4. Valid 4Cseq reads containing 4C reading primer were extracted from fastq file and parsed into raw .txt file aligned against the restriction-enzyme digested genome GRCh37(hg19) using 4Cseqpipe version 0.7 (van de Werken et al. 2012).<br>Raw files were translated into final graphical depictions of contact profiles around viewpoints, using 4Cseqpipe version 0.7 (van de Werken et al. 2012).

For manuscripts utilizing custom algorithms or software that are central to the research but not yet described in published literature, software must be made available to editors and reviewers. We strongly encourage code deposition in a community repository (e.g. GitHub). See the Nature Research guidelines for submitting code & software for further information.

Data

Policy information about availability of data

All manuscripts must include a data availability statement. This statement should provide the following information, where applicable:

- Accession codes, unique identifiers, or web links for publicly available datasets
- A list of figures that have associated raw data
- A description of any restrictions on data availability

hg19 reference genome is available from NCBI (GenBank assembly accession: GCA_000001405.1).
hg38 reference genome is available from NCBI (GenBank assembly accession: GCA_000001405.15).
The deleted sequences information is available at Decipher database (#411659).
ChIP-seq and 4C-seq data are available at the GEO website (Accession number: GSE155324).

Field-specific reporting

Please select the one below that is the best fit for your research. If you are not sure, read the appropriate sections before making your selection.

[x] Life sciences [ ] Behavioural & social sciences [ ] Ecological, evolutionary & environmental sciences

For a reference copy of the document with all sections, see nature.com/documents/nr-reporting-summary-flat.pdf

Life sciences study design

All studies must disclose on these points even when the disclosure is negative.

Sample size	No statistical method was used to pre-determine the sample size for the animal experiments. Sample size was determined to obtain reproducibility and reliable distribution among biologically independent samples, based on extensive laboratory experience and literature in this field (Paliou et al. PNAS (2019) PMID: 31147463, Williamson et al. Development (2019) PMID: 31511252). Sample sizes are described in the manuscript.
Data exclusions	No data were excluded from analyses.
Replication	For ChIP-seq and 4C-seq, two technical replicates were used. All attempts were successful. All replicate data are shown in Fig S5 and S6.
Randomization	Control wild type and knockout mice used for analysis were chosen randomly.
Blinding	Sample preparation, sequencing and computational analysis were performed by different researchers. The investigators were not blinded during sample preparation due to feasibility of experiments. For computational analysis, sample names were blinded until the final outputs were generated.

Reporting for specific materials, systems and methods

We require information from authors about some types of materials, experimental systems and methods used in many studies. Here, indicate whether each material, system or method listed is relevant to your study. If you are not sure if a list item applies to your research, read the appropriate section before selecting a response.

Materials & experimental systems

n/a	Involved in the study	
[ ]	[x]	Antibodies
[ ]	[x]	Eukaryotic cell lines
[x]	[ ]	Palaeontology and archaeology
[ ]	[x]	Animals and other organisms
[ ]	[x]	Human research participants
[x]	[ ]	Clinical data
[x]	[ ]	Dual use research of concern

Methods

n/a	Involved in the study	
[ ]	[x]	ChIP-seq
[x]	[ ]	Flow cytometry
[x]	[ ]	MRI-based neuroimaging

Antibodies

Antibodies used	CTCF (Active Motif, catalog no. 61311)<br>RAD21 (Abcam, catalog no. ab992)<br>Six µg of antibody was used for the binding with magnetic beads (final concentration was 60ng/µL in the binding reaction).
Validation	All antibodies have been extensively used in other studies:

CTCF: Rodríguez-Carballo, Eddie, et al. "The HoxD cluster is a dynamic and resilient TAD boundary controlling the segregation of antagonistic regulatory landscapes." Genes & development 31.22 (2017): 2264-2281.
RAD21: Schwarzer, Wibke, et al. "Two independent modes of chromatin organization revealed by cohesin removal." Nature 551.7678 (2017): 51-56.

Eukaryotic cell lines

Policy information about cell lines

Cell line source(s)	Lymphoblastoid cells were from the proband, parents.<br>WT lymphoblastoid cells were a kind gift from Dr. Jorge Oksenberg at UCSF.
Authentication	The study was approved by the ethical committee of the University of California San Francisco, protocol number 10-03111, Comitê de Ética em Pesquisa da Prefeitura de Porto Alegre (Plataforma Brasil) protocol number 1.103.654 and the Brazilian Research Ethics Commission (CONEP) protocol number 223.811. Samples were obtained after receipt of informed consent.
Mycoplasma contamination	Not tested.
Commonly misidentified lines<br>(See ICLAC register)	No commonly misidentified cell lines were used.

Animals and other organisms

Policy information about studies involving animals; ARRIVE guidelines recommended for reporting animal research

Laboratory animals	FVB (Jackson Laboratory, catalog no. 001800) was used for all experiment.<br>Animals were used for the experiment at embryonic day(E)11.5 or E18.8 as described in the figure legends.<br>Both male and female were used for analysis.<br>All animals were maintained in a temperature-controlled room (temperature 19.4- 23.3 °C , humidity 30-70%) on a 12 h light/dark cycle with light onset at 7am.<br>LMBR 12 kb deletion allele was generated by iGONAD method. Please see "Generation of knockout mice" section in manuscript.
Wild animals	No wild animals were used in this research.
Field-collected samples	No field-collected samples were used in this research.
Ethics oversight	Mouse work was approved by the UCSF IACUC, protocol number AN100466, and was conducted in accordance with AALAC and NIH guidelines.

Note that full information on the approval of the study protocol must also be provided in the manuscript.

Human research participants

Policy information about studies involving human research participants

Population characteristics	The acheiropodia family (parents and affected individual) were recruited and selected for this study due to the acheirpodia phenotype that the affected individual has.
Recruitment	The family was recruited during medical examination and all samples and photos were obtained after receipt of informed consent.
Ethics oversight	The study was approved by the ethical committee of the University of California San Francisco, protocol number 10-03111, Comitê de Ética em Pesquisa da Prefeitura de Porto Alegre (Plataforma Brasil) protocol number 1.103.654 and the Brazilian Research Ethics Commission (CONEP) protocol number 223.811.

Note that full information on the approval of the study protocol must also be provided in the manuscript.

ChIP-seq

Data deposition

- [x] Confirm that both raw and final processed data have been deposited in a public database such as GEO.
- [x] Confirm that you have deposited or provided access to graph files (e.g. BED files) for the called peaks.

Data access links<br>*May remain private before publication.*	ChIP-seq and 4C-seq data are available at the GEO website. Accession number is GSE155324.
Files in database submission	GSM4699144 CTCF-ChIP-seq-Mut-rep1<br>GSM4699145 CTCF-ChIP-seq-INPUT-Mut-rep1<br>GSM4699146 CTCF-ChIP-seq-Mut-rep2<br>GSM4699147 CTCF-ChIP-seq-INPUT-Mut-rep2<br>GSM4699148 CTCF-ChIP-seq-WT-rep1

GSM4699149 CTCF-ChIP-seq-INPUT-WT-rep1
GSM4699150 CTCF-ChIP-seq-WT-rep2
GSM4699151 CTCF-ChIP-seq-INPUT-WT-rep2
GSM4699152 RAD21-ChIP-seq-Mut-rep1
GSM4699153 RAD21-ChIP-seq-INPUT-Mut-rep1
GSM4699154 RAD21-ChIP-seq-Mut-rep2
GSM4699155 RAD21-ChIP-seq-INPUT-Mut-rep2
GSM4699156 RAD21-ChIP-seq-WT-rep1
GSM4699157 RAD21-ChIP-seq-INPUT-WT-rep1
GSM4699158 RAD21-ChIP-seq-WT-rep2
GSM4699159 RAD21-ChIP-seq-INPUT-WT-rep2
GSM4699160 4C-seq-SHH-viewpoint-WT-rep1
GSM4699161 4C-seq-SHH-viewpoint-WT-rep2
GSM4699162 4C-seq-SHH-viewpoint-Mut-rep1
GSM4699163 4C-seq-SHH-viewpoint-Mut-rep2

Genome browser session (e.g. UCSC)

Not available.

Methodology

Replicates

Two biological replicates.

Sequencing depth

30-50M reads.

Antibodies

CTCF (Active Motif, catalog no. 61311)
RAD21 (Abcam, catalog no. ab992)

Peak calling parameters

The ChIP-seq raw reads were mapped against the hg19 with bowtie2(2.2.6) , duplication were removed with Picard (1.126) MarkDuplicates and peaks were called using MACS2(v2.1.1 -p 1e-2 --nomodel --shift 0 --keep-dup all -B --SPMR), Reproducible peaks were identified by IDR(2.0.4) at a threshold of 5%.Then the differential enrichment analysis between Mutation and Wildtype is performed by DiffBind(3.11)

Data quality

Number of peaks called:
CTCF-Mut_rep1 61241
CTCF-Mut_rep2 42333
CTCF-Mut_pool 61995
CTCF-WT_rep1 39590
CTCF-WT_rep2 35961
CTCF-WT_pool 43634
RAD21-Mut_rep1 62861
RAD21-Mut_rep2 43189
RAD21-Mut_pool 50140
RAD21-WT_rep1 59137
RAD21-WT_rep2 62438
RAD21-WT_pool 57879

Software

MACS2(v2.1.1), bowtie2(2.2.6), IDR(2.0.4), SAMtools(1.7),Picard (1.126), bedtools (2.26.0), IDR(2.0.4), DiffBind(3.11)
